# A multilayer drift‐diffusion broadcast model: Converting temporal evidence into response timing

**DOI:** 10.1002/jeab.70150

**Published:** 2026-10-26

**Authors:** Fuat Balcı

**Affiliations:** ^1^ Department of Biological Sciences University of Manitoba Winnipeg MB Canada

**Keywords:** computational model, drift diffusion, interval timing, leaky integration, peak interval procedure

## Abstract

The peak interval procedure, in which subjects learn to cluster their anticipatory responses around delays to biologically critical outcomes, provides benchmarks for interval timing models. These include (a) the bistable response regime demarcated by an abrupt increase (start times) and a decrease (stop times) in response rates around the target time in individual trials; (b) bell‐curve‐shaped average response curves that superpose on a relative time scale; (c) lower coefficient of variation of stop times than start times; and (d) specific correlational patterns between these parameters, their mean, and difference. This article presents a multilayer computational model that predicts these key markers of timing behavior in the peak interval procedure. The first layer constitutes the clock stage, comprising uncoupled diffusion processes approaching a single boundary. The first crossing of the threshold for each diffusion process is broadcast to the downstream motor layer. The motor layer integrates the broadcast signals with a leak term scaled by the drift rate to avoid saturation and reset of motor system activity. The model is extended to account for real‐time temporal discrimination. This family of models departs from the single‐clock approach to modeling and meets the core empirical benchmarks of timing behavior.

## INTRODUCTION

Animals keep track of time intervals ranging from seconds to minutes using an evolutionarily well‐preserved internal clock, referred to as interval timing. Interval timing manifests as the modulation of organisms' anticipatory actions such that outcome‐oriented actions cluster around the time of that outcome, depending on its temporal relationship to preceding events. The timing behavior readily emerges in experimental settings where the reward becomes available reliably after a fixed delay following the preceding event (e.g., fixed‐interval (FI)/fixed‐time (FT) schedules). For example, when the reward becomes available 10 s after the onset of a tone, animals initiate their anticipatory responses 5 s after the predictive stimulus begins (Church et al., 1994; see also Pavlov, 1927 for similar observations in the classical conditioning). When this delay increases to 24 s, the same animals may now initiate their responses after 12 s, indicating timescale invariance in interval timing (i.e., the overlap of timing behaviors on a relative timescale; Gibbon, 1977). The interval timing also enables the animal to stop anticipating this reward when it is omitted, akin to realizing that the traffic light is broken when it is indeed stuck on red.

To investigate interval timing in such scenarios, Catania (1970) and Roberts (1981) developed the peak interval (PI) procedure. In the PI procedure, animals are typically first trained to anticipate the reward following a FI after the onset of a predictive stimulus (FI trials), allowing animals to learn the delay between the timing stimulus and outcome. However, responses during FI trials capture only the first half of the timing behavior due to reward delivery. To capture the interval timing behavior in its entirety, PI trials are intermixed with FI trials in which the predictive stimulus lasts much longer than the FI trial and the reward is omitted. In an individual PI trial, subjects begin responding as the reward delivery time approaches and stop responding sometime after the reward is omitted, resulting in a break‐run‐break pattern in which the run period contains the reward delivery time (Cheng & Westwood, 1993; Church et al., 1994; Gibbon & Church, 1990; Gibbon et al., 1984; Sanabria et al., 2009).

The response initiation and termination times that demarcate the run period are called the start time and stop time, respectively. The start and stop times are typically positively correlated; the start times and spread (stop time–start time) are typically negatively correlated, and the middle times, (start time + stop time) / 2), and spread are weakly positively correlated or not correlated (Gibbon & Church, 1990; Luzardo et al., 2017). However, Garces et al. (2018) and Lejeune et al. (1998) have also reported negative middle‐time–spread correlations. Furthermore, the stop times have a lower coefficient of variation than the start times (Balcı & Freestone, 2020; Luzardo et al., 2017). A bell‐curve‐shaped response curve emerges when the response times are averaged across PI trials, with its peak corresponding to the reward delivery time. Crucially, the above‐mentioned response patterns in the PI procedure have served as empirical benchmarks for many computational models of interval timing (Balcı & Freestone, 2020; Gibbon & Church, 1990; Killeen & Fetterman, 1988; Killeen & Taylor, 2000; Luzardo et al., 2017).

The early models were developed within the framework of scalar timing theory (SET: Gibbon, 1977; Gibbon et al., 1984), a pacemaker‐accumulator model of interval timing (Balcı & Simen, 2016, 2024). Specifically, SET assumes that clock signals are generated by a Poisson process (i.e., the clock's intertick time is an exponentially distributed random variable) and counted by an accumulator. When the reward is delivered, the total number of clock ticks integrated by the accumulator is consolidated into long‐term memory. Importantly, this consolidation process is assumed to be perturbed by an unbiased, normally distributed random variable: $t_{\mathrm{LTM}}$= $N\left(1,\sigma \right)\cdot \hat{t}$. This translation process yields an unbiased but noisy, timescale‐invariant long‐term memory representation of time intervals. In PI trials, a decision module compares the number of ticks in the accumulator $\hat{t}$ to a random sample from the long‐term memory $\hat{t}$_*LTM*_ according to a ratio rule $\left(\left|\;\hat{t}-{\hat{t}}_{\mathrm{LTM}}\right|/\;{\hat{t}}_{\mathrm{LTM}}\right)$. The animal responds in anticipation of reward while the value of this decision variable remains below a certain level (effectively a decision threshold), yielding the break‐run‐break pattern. Importantly, the variability at the level of LTM predicts a positive correlation between the start and stop times, a negative correlation between the start time and spread, and a positive correlation between the middle time and spread observed in empirical data (Gibbon & Church, 1990) and is the primary source of scalar variability in timing behavior (Balcı & Freestone, 2020; Gibbon et al., 1984). This model also predicts lower coefficients of variation (CV) for stop times than start times.

Other models predict these empirical benchmarks to different degrees. To this end, Peter Killeen and Greg Fetterman's seminal behavioral theory of timing (BET; Killeen & Fetterman, 1988) asserts that temporal differentiation is embedded in the hypothetical behavioral states and the between‐state transitions (as for temporal‐difference learning algorithm; Sutton & Barto, 1998). BET assumes that the between‐state transition rate scales inversely with the target interval (e.g., proportional to the reward rate, Reward_Magnitude_ / Reward_Delay_). Since the reward would be delivered reliably and coincidentally with a given state, being in the corresponding state becomes a discriminative stimulus (*S*_*D*_) eliciting the anticipatory response. Crucially, through stimulus generalization, albeit at a lower level, the neighboring states would also gain predictive power regarding reward delivery, thereby eliciting a more limited degree of anticipatory responses (resulting in a generalization gradient around the target time). The switch from a break to a run (start time) corresponds to entering the adjunctive active states in the chain, and the switch from a run to a break (stop time) corresponds to entering the adjunctive inactive state in the chain. BET is also successful at predicting average response curves.

Evolving directly from BET, the learning to time (LeT) model of Armando Machado (1997; Machado et al., 2009) assumed that the states that were active at the time of reward delivery earn an associative weight with the response module proportionally to their activity level during reward delivery. Thus, the associative gain is lower for states neighboring the most active state but are less active at the time of reward delivery. The probability of a response is directly proportional to the associative strength of the currently active states, as with BET, although LeT successfully captures the average response curve and the break‐run‐break response pattern in individual trials when the associative weights are subjected to a behavioral manifestation threshold.

Unlike these models of interval timing, the striatal beat‐frequency model (Matell & Meck, 2004) assumes that the basis functions for timed actions are cortical oscillatory clocks with different frequencies (see also Varella et al., 2019). These oscillations are assumed to phase‐reset via mesocortical phasic dopamine signaling contingent on the onset of the predictive stimulus. The medium‐spiny neurons of the dorsal striatum receive synaptic inputs from a specific subset of these cortical oscillators and strengthen synaptic connections only to those oscillations that happen to fire simultaneously at the time of reward delivery (through reward‐related nigrostriatal dopamine signaling). Thus, the medium‐spiny neurons act as coincidence detectors when the specific oscillatory alignment matrices match previously learned patterns. Introducing a firing threshold for medium‐spiny neurons can approximate the break‐run‐break pattern. However, an implausibly massive and highly dedicated pool of cortical inputs is needed (e.g., thousands of cortical inputs per striatal neuron; Oprisan & Buhusi, 2013, 2014) to achieve stable, precise interval timing, which requires a substantial resource commitment just for the timing function itself. Furthermore, the resulting neuronal representation of time intervals lacks the metric information preserved in animals' temporal representations (Drew & Balsam, 2001; Gibbon & Church, 1981).

Another pacemaker‐accumulator family of interval timing models, in general, and PI responding in particular, emerges as a variant of Ratcliff's drift‐diffusion model successfully applied to perceptual decision making for decades (Ratcliff, 1978; Ratcliff & McKoon, 2008; Ratcliff & Rouder, 1998)—namely, the time‐adaptive opponent‐Poisson drift‐diffusion model (TopDDM; Balcı & Simen, 2016; Simen et al., 2011, 2013). TopDDM assumes that a drift‐diffusion process with a single threshold (*z*) in which the drift is inversely proportional to the target interval (A=z/T) and the diffusion coefficient is proportional to the square root of the drift rate ($\sqrt{A}$) captures the core statistical features of interval timing, including the timescale invariance. Effectively, as a clock‐adaptive model, Peter Killeen's BET (Killeen & Fetterman, 1988) motivated yet another model of interval timing (i.e., TopDDM).

However, the original formulation of the TopDDM is not readily applicable to PI timing. For example, in TopDDM the height of that single threshold (*z*) is fixed at the maximum possible limit to filter out diffusion noise and optimize temporal precision. This poses a challenge for modeling to generate the break‐run‐break pattern with a single clock (unless its threshold crossing is assumed to trigger a Gaussian radial basis function—for example, Luzardo et al., 2017—subjected to a threshold, which fails to account for the empirical benchmarks [unpublished simulations]). Instead, it results in a point‐timed response for a single drift‐diffusion process and approximates inverse‐Gaussian‐distributed threshold first‐crossing times for multiple parallel processes. Luzardo et al. (2017) sought to force a single‐stage accumulator to produce a “break‐run‐break” pattern by applying two distinct thresholds to the same accumulator: a lower start boundary (*z*_start_) and an upper stop boundary (*z*_stop_).

This model captures the critical statistical signatures of behavioral timing data, including the correlational patterns and the lower CV of stop times than of start times; however, the double‐threshold assumption does not align well with the conceptual logic of TopDDM's original formulation. Including two thresholds instead of one makes the lower threshold relatively more vulnerable to early stochastic noise fluctuations than the highest possible timing threshold (which also results in a higher start time CV). This violates the high‐precision, noise‐filtering advantage of TopDDM's high ceiling. Another resulting violation of the assumptions of the TopDDM is that the accumulation process continues after crossing a threshold (i.e., the lower start threshold).

More recently, Zwicker and Rivest (2022) developed a threshold‐free variant of TopDDM (probabilistic response drift–diffusion model [PRDDM]) to capture responding in the PI procedure. In this model, the probability of response emission varies over time (following a linear relation between the clock state and response probability), depending on the time‐dependent likelihood of reward delivery—namely, a stochastic response mechanism driven directly by noise in the internal clock. This model captures the core signatures of PI responding but suffers from a mechanistic gap regarding how time‐dependent likelihoods are converted to responses.

All these models assume that a single clock process is in effect in each trial (although this assumption is implicitly broken in the striatal beat‐frequency model). However, pacemaker‐accumulator models relying on a single, centralized master pacemaker require a high degree of local neural modularity to protect the accumulating trace from compounding stochastic noise, particularly over extended durations—a requirement that clashes with the highly noisy, uncoupled nature of individual cortical neurons. By operationalizing temporal tracking through a population of parallel, uncoupled processes would instead mirror the “population clock” dynamic frequently observed in corticostriatal circuits, where time is decoded from distributed, evolving state–space trajectories (e.g., Gershman, Moustafa, et al., 2014; Staddon & Higa, 1996). A multiple‐clock framework also provides two key biological and functional benefits. First, it introduces structural robustness simply through redundancy because by relying on the averages of pooled inputs, the system degrades “gracefully” under process loss or localized neural degradation (akin to the degeneracy idea introduced by Turgeon et al., 2016) rather than experiencing total failure. Second, by pooling across multiple stochastic channels, the architecture effectively leverages the law of large numbers to cancel out individual, microlevel noise. This allows the nervous system to construct stable macrolevel behavioral momentum from inherently noisy microlevel cellular components.

In contrast to the basic and often implicit assumption of a single clock, Hasegawa and Sakata (2015) assumed the simultaneous operation of independent clocks and a mixer component that integrates the weighted average of the overlapping outputs from these clocks, which is then relayed to the memory and response modules. However, this model did not address the single‐trial response patterns. Furthermore, it effectively combined SET and LeT, introducing additional computational layers and complexity to account for responses in the PI procedure, and relied heavily on the mixer component's complex integration operation. That said, Hasegawa and Sakata stands out in the conceptual scenery because it is not constrained to a single clock mechanism and multiple independent clock mechanisms indeed appear necessary to account for higher order interval‐timing functions such as retrospective and prospective temporal error monitoring (Akdoğan & Balcı, 2017; Balcı, 2022; Balcı & Öztel, 2026) and temporal averaging (De Corte & Matell, 2016).

This article introduces a new model of timing behavior in the PI procedure that relies on TopDDM without violating its computational and conceptual principles (i.e., maximally set single threshold). Unlike many other models, it relies on multiple independent clocks and mechanistically maps their operation onto responses, based on widely accepted neural dynamics such as leaky integration. In this sense, the current model more effectively complements and integrates with the TopDDM framework by introducing new, neurally acceptable information‐processing layers that translate temporal information into temporally guided anticipatory actions in real time. The model's behavior is compared with the critical benchmarks derived from the literature (e.g., Gibbon & Church, 1990; Luzardo et al., 2017).

## METHODS

### Model specification

The *multi‐layer drift‐diffusion broadcast model* (ML‐TopDDM) architecture is composed of an upstream clock layer (i.e., TopDDM) that broadcasts clock outputs—that is, threshold (*z*) first crossings of diffusion processes, *x*(*t*)—to a downstream motor layer, where these signals are integrated in real time with leak, *Y*(*t*), and subject to a response threshold (θ) to translate their activity level to anticipatory responses (Figure 1, first and second panels).

**FIGURE 1:**
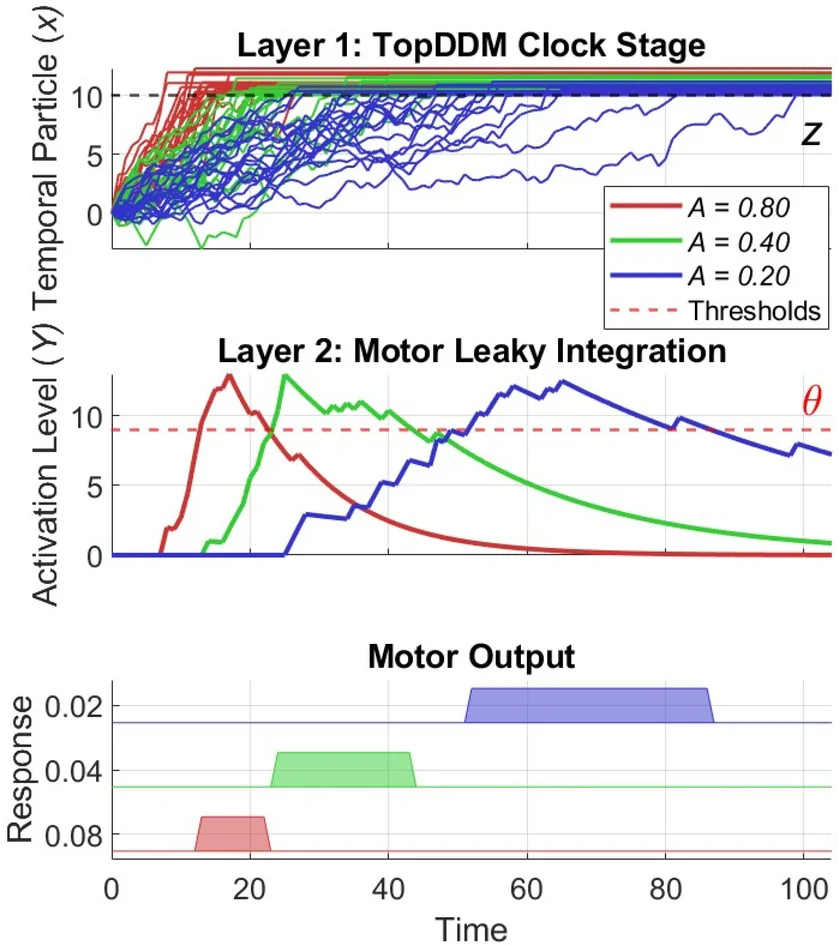
This illustration shows the reduced model's three‐layered states. The top row shows the clock layer with TopDDM‐based diffusion processes for three target intervals (each target is implemented by operating 20 independent clocks). Note that a few cases in which the diffusion process falls below 0 do not lead to large deviations from the distributional assumptions. The middle row shows the motor layer with the activity level of the leaky integration separately for three different target intervals. Each input to the leaky motor integration corresponds to a threshold crossing at Layer 1 for the corresponding target interval. The horizontal dashed line in the middle row is the response threshold; when the leaky integrator's activity exceeds this threshold, the timing agent responds in anticipation of the reward, resulting in a break‐run‐break pattern of responses in individual trials (bottom row).

#### Clock layer

TopDDM assumes that the internal clock relies on balanced excitation and inhibition, with inhibitory Poisson inputs being a constant proportion of excitatory Poisson inputs. The input current (*I*) provided to this competitive integration process is inversely proportional to the target time (inspired by Peter Killeen's BET's logic; Killeen & Fetterman, 1988), thereby constituting TopDDM's clock‐adaptive property $I\cdot \left(1-\gamma \right)$. The variance of this competitive Poisson process is the sum of the variances of both processes per Skellam distribution (Skellam, 1946), which is equal to the sum of the rates of each Poisson input ($c^{2}=I\cdot \left(1+\gamma \right)$; variances add up despite subtraction), resulting in the following diffusion process (Equation 1):

(1) $$\mathrm{dx}=I\cdot \left(1-\gamma \right)\cdot \mathrm{dt}+\sqrt{\left(1+\gamma \right)\cdot I}\cdot \mathrm{dW}.$$

When the drift rate is $A=I\cdot \left(1-\gamma \right)$ and we define $m=\sqrt{\left(1+\gamma \right)/\left(1-\gamma \right)}$, substitution into the equation above with $c=\sqrt{\left(1+\gamma \right)\cdot I}$ results in $c=\sqrt{\left(1+\gamma \right)/\left(1-\gamma \right)\cdot A}$. After getting $\sqrt{\left(1+\gamma \right)/\left(1-\gamma \right)}$ out of the equation, *c* becomes $m\sqrt{A}$, as shown in Equation 2:

(2) $$\mathrm{dx}=A\cdot \mathrm{dt}+m\cdot \sqrt{A}\cdot \mathrm{dW},$$

where the diffusion standard deviation is proportional to the square root of the drift. This property is necessary for ensuring timescale invariance of the first‐crossing times under a constant single threshold (*z*) and an adaptive clock (*A*). Crucially, the threshold first‐crossing times of a drift‐diffusion process are inverse‐Gaussian distributed, as observed in empirical data with a skewness/CV ratio of three for the manifestation of the Skellam process as drift diffusion (CV being $\sqrt{\mu /\lambda }$ and skewness being $3\sqrt{\mu /\lambda }$). Note that BeT (Killeen & Fetterman, 1988) becomes a special case of TopDDM when γ is 0 (Simen et al., 2013), resulting in a skewness/CV ratio of two (CV being $1/\sqrt{ɑ}$ and skewness being $2/\sqrt{ɑ}$).

Critically, the ML‐TopDDM assumes that instead of running a single clock, multiple uncoupled, independent drift‐diffusion processes with the same drift rate (*A*) that are set to hit the threshold at the target time (*T*) operate in parallel in a single trial. Due to the diffusion coefficient, each clock hits the timing threshold at different times (on average at *T*), where *t*_*i*_ is the first time at which an individual process *x*_*i*_ crosses the threshold (*z*), as shown in Equation 3.

(3) $$x_{i}\left(t_{i}\right)\geq z,$$

where $t_{i}>0$ is the unique time of threshold crossing for channel *i*. The top row of Figure 1 shows the sample TopDDM processes (20 independent processes per *T*).

#### Motor layer

The threshold crossing of each of these diffusion processes is broadcast to a downstream motor leaky integrator with its own threshold (θ), subject to leakage proportional to the drift rate (λ=α·A; e.g., slower leak for slower clock). The dynamics of the motor‐layer computations *Y*(*t*) are governed by a leak rate λ. When the drift rate is high, the integrator has a proportionally strong leak, causing its activity to decay toward baseline when it is not continuously driven. This makes the motor system rely on highly reliable, sustained clock signals when the target interval is relatively short. With a low λ, the downstream leaky integrator of high‐drift‐rate outputs would saturate due to overaccumulation and, as a result, lose temporal precision (a form of information clipping or loss of dynamic range). When the drift rate is low, the leaky integrator can integrate sparse (spread out) and weak inputs over longer time windows (with less forgetting during periods of “clock silence”). If the λ is too high (short memory window 𝜏), the motor integrator will effectively lose the memory of early crossings of slow drift‐diffusion processes before the arrival of subsequent ones and, as a result, may never register the timer population event. Thus, the downstream motor leaky integrator must adapt its memory window (𝜏) to match the rate of broadcast clock inputs from Layer 1. The above‐mentioned dynamics of the motor layer leaky integrator can be formulated as follows in Equation 4:

(4) $$\frac{\mathrm{dY}\left(t\right)}{\mathrm{dt}}=\omega \sum_{i=1}^{N}\delta \left(t-t_{i}\right)-\alpha A\cdot Y\left(t\right)$$

(subject to Equation 3), where *N* is the number of independent TopDDMs, *Y*(*t*) is the internal state of the motor layer leaky integrator at time *t*, α is the positive scaling constant mapping the leak onto the drift rate (*A*), ω is the step increase in *Y* by a clock signal broadcast from Layer 1 (which can be set to 1), and *t*_*i*_ is the time of the threshold first crossing of the *i* ^th^ TopDDM (Equation 3). When the motor layer's leaky integrator activity exceeds the fixed‐response threshold, the animal enters a state of anticipation and begins responding for reward: $\text{Response}\left(t\right)=\left\{\begin{array}{c} 1,\text{if}\;Y\left(t\right)\geq \theta \\ 0,\text{if}\;Y\left(t\right)<\theta \end{array}\right.$.

The leak component causes the motor integrator's activity level to fall below the same response threshold sometime after (mostly) the last threshold crossing, thereby terminating the animal's anticipatory period, as marked at the behavioral level by the cessation of anticipatory responding (bottom panel of Figure 1). The threshold first‐crossing time of the leaky integrator's activity marks the start time, and the last time this activity goes below the response threshold marks the stop time. The middle row of Figure 1 shows the activity state of leaky accumulation for three different target intervals, along with the common response threshold, θ. The bottom row of Figure 1 illustrates the sample behavioral break‐run‐break patterns for three different target intervals. Figure 2 presents the flowchart diagram of ML‐DDM and its extensions. Table 1 lists the model parameters along with their computational role and theoretical meaning.

**FIGURE 2:**
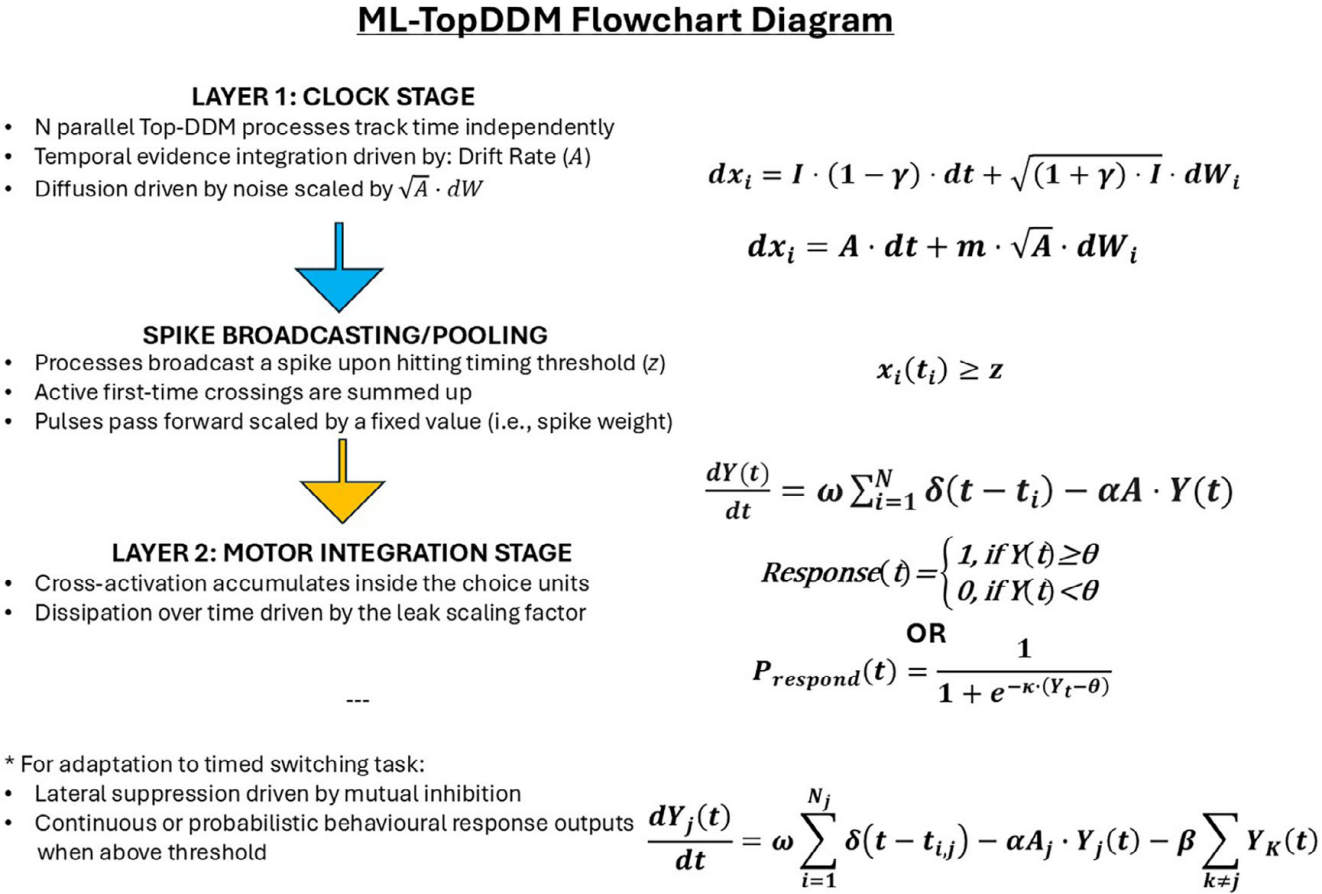
The flowchart formally outlines the functional architecture of the models presented in the current article.

**TABLE 1:** List of model parameters along with their computational role and theoretical meaning.

Parameter name	Script variable	Tested values	Computational role and theoretical meaning
Drift rate (*A*)	drift_rates	.2, .4, .8	Dictates the slope of temporal evidence integration in Layer 1. Negatively correlated with the target interval.
Scaled diffusion (*m*)	*k*_sigma	1.225 (Figure 3, Figure 4) 1.527 (Figure 5)	Stochastic variable: Scales diffusion as a function of $\sqrt{A}$. Affects the level of timing variability.
Clock threshold (*z*)	*k*_threshold	10	The threshold for Layer 1: Determines how much evidence an individual diffusion process requires to freeze and broadcast to Layer 2.
Spike weight (ω)	spike_weight	1	Fixed at unity: Dictates the driving force of a single clock crossing broadcast on the motor layer.
Leak factor (α)	*k*_leak	.1	Inherent decay constant: Scales the leaky decay of the motor unit relative to the drift rate. Critical to achieve the scalar property at the level of behavioral outputs.
Motor threshold (θ)	integrator_threshold [Reduced model parameter]	9	Response threshold of Layer 2 for motor activation: Responses are deterministically [on/off switch] or probabilistically emitted and sustained while the motor unit activity remains above this threshold or *p* = .50 responding for the probabilistic case.
Steepness (κ)	*k*_steepness [Extended model parameter]	1.5	The steepness of the logistic function that maps the response probability onto the activity level of the motor layer.
Competition weight (β)	*k*_competition [Switch task version]	.01	Lateral inhibitory constant: Determines the strength of mutual cross‐suppression. Multiplied by the competitor activation to generate a linear downward force.

In its simplest form, ML‐DDM has four free architectural parameters. The first two free parameters are inherited directly from the TopDDM architecture. These represent the diffusion scaling parameter, which reflects the proportion of inhibitory input relative to excitatory input, and the timing threshold parameter. The other two free parameters that belong to the new layer are the leak rate and the motor threshold.

### Numerical simulations

The simulations of the model outlined above were conducted in MATLAB over 500 runs with the following parameter values. The number of TopDDM timers manifested per trial was initially set to 20 (*N* = 20). The drift rates were [0.80, 0.40, 0.20] for three different intervals, and *dt* was set to 1. The base‐level diffusion noise was set to 1.225 (corresponding to a γ value of .20) and then scaled by the square root of the drift rate to implement the TopDDM process. The timer threshold was set to 10, and the leaky integrator's response threshold to 90. The value of ω was set to 1. The run‐ready numerical simulation codes for all models are available in the Supplemental Materials.

## RESULTS

Figure 3 shows the average response curves for three different target intervals, estimated using the simplest form of ML‐DDM with on–off switch logic. The visual inspection of Figure 3 shows the widening of the average response curves across three hypothetical target intervals, with near‐perfect superposition on the relative timescale. This observation provides very strong support for the resultant scalar property.

**FIGURE 3:**
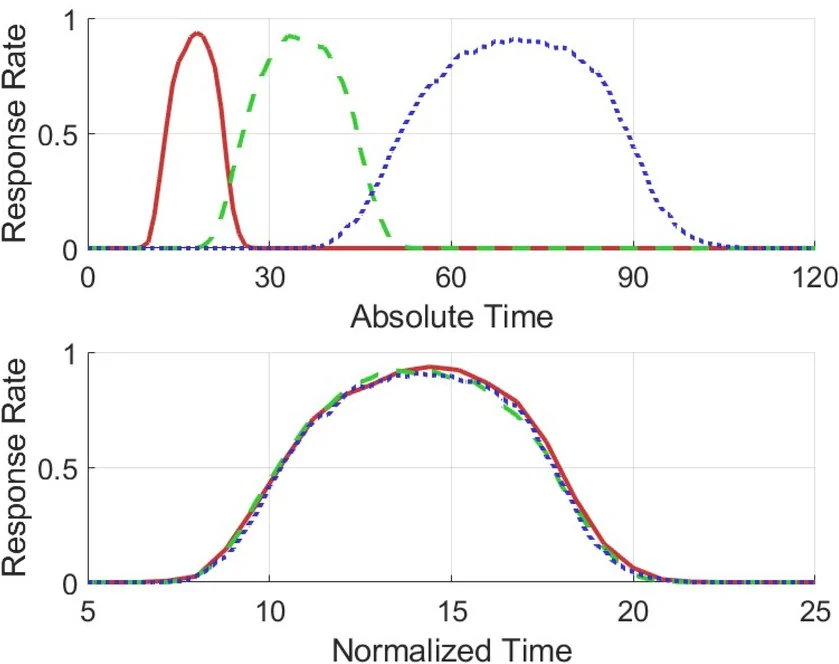
The *top row* shows the average response rate (averaged across trials, each characterized by the break‐run‐break response pattern) for three different intervals. The visual inspection of these curves shows that they widen as the target interval increases. The *bottom row* shows the superposition of the average response curves when expressed on a relative timescale.

The emergent correlational patterns were largely consistent with those reported in Gibbon and Church (1990; Figure 4 in the current article; except for the one case of statistically significant middle–spread negative correlation). There was a significant positive start–stop correlation for all target intervals (*r* = .46, .49, and .48, respectively, all *p* = .0001, Bonferroni corrected for nine comparisons). There was a significant negative start–spread correlation for all target intervals (*r* = −.50, −.62, and −.57, respectively, all *p* = .000, Bonferroni corrected). There was a significant middle‐time–spread negative correlation only for the second target interval (*r* = −0.19, *p* = .0001, Bonferroni corrected). The correlation coefficients were −.11 and −.09 for the short and long intervals, respectively. Figure 5 illustrates the correlational patterns gathered by simulating the reduced ML‐DDM. The CVs of start times (.14, .14, and .14) were consistently higher than those for stop times (.079, .073, and .074, respectively). The near‐constant start‐ and stop‐time CVs across different durations also supported the scalar property (in addition to the superimposition of the average response curves).

**FIGURE 4:**
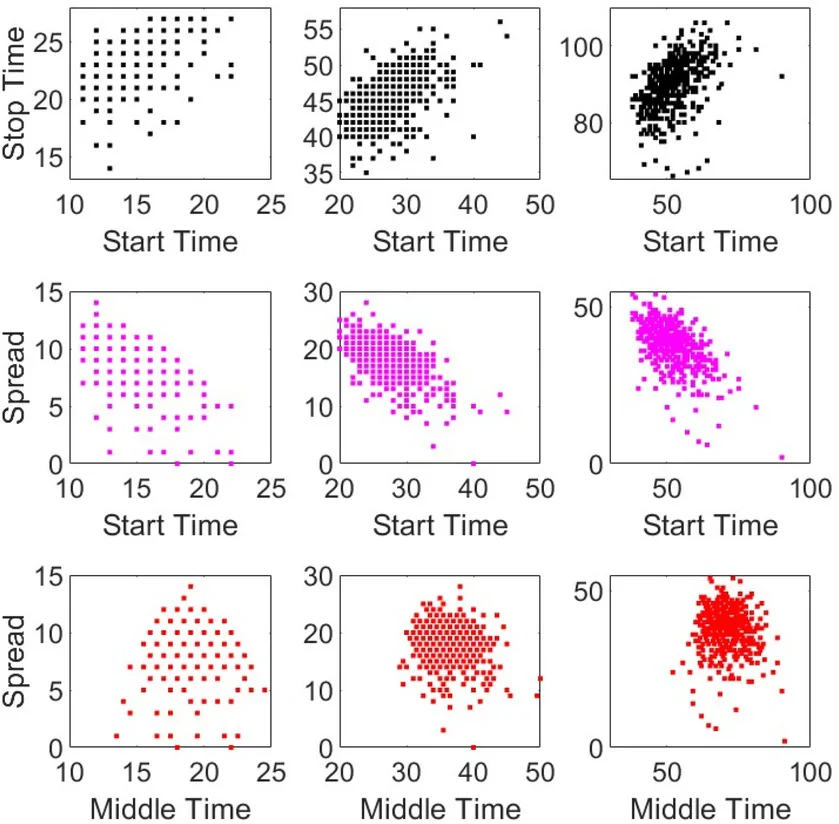
The start–stop, start–spread, and middle‐time–spread scatter plots gathered from the simulations of the reduced ML‐DDM, separately for three different intervals.

**FIGURE 5:**
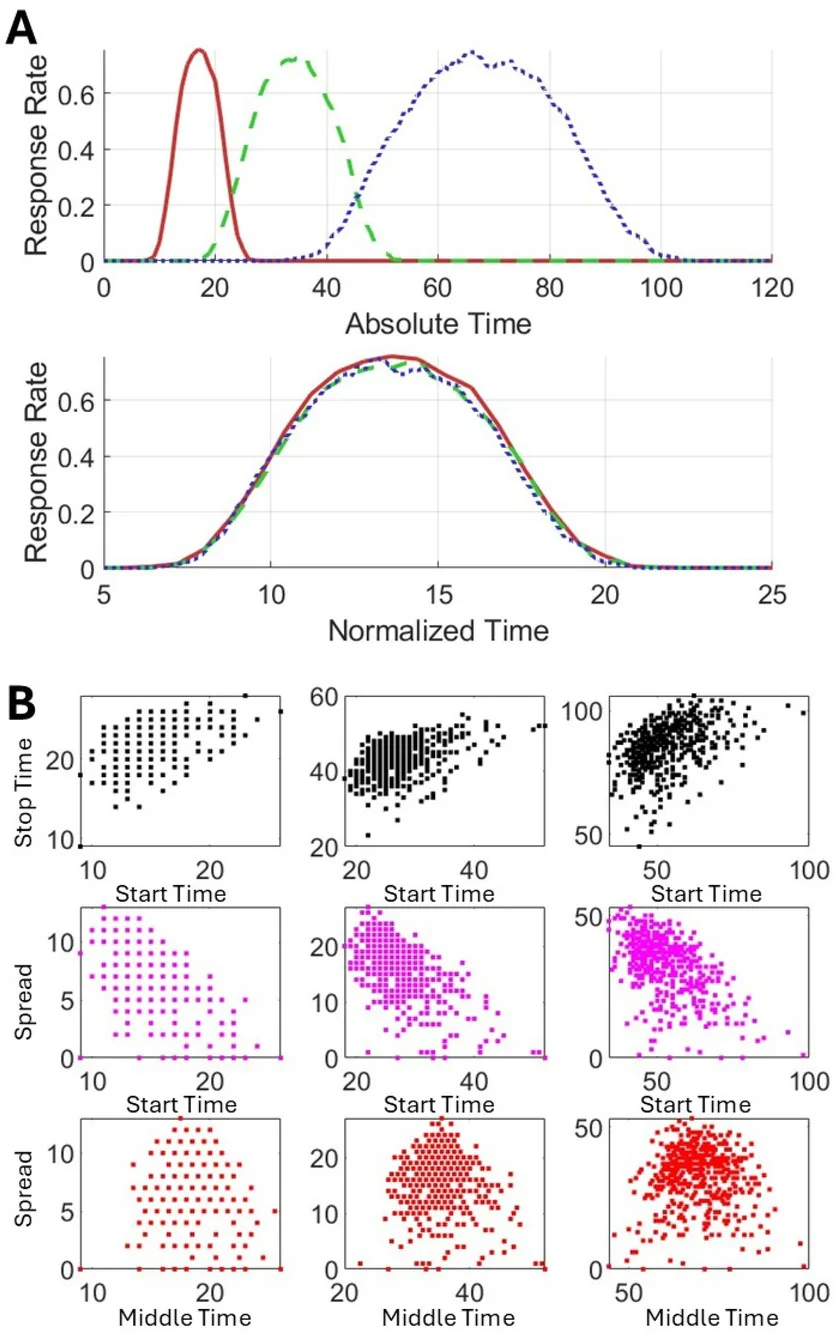
Results of simulations conducted with a diffusion scaling of 1.527. Panel A shows the gathered average response curves on both absolute and relative timescales. Panel B shows the scatter plots that correspond to three key correlation patterns.

The same simulations were conducted with a diffusion scaling factor of 1.527 (corresponding to .40 inhibitory input per one excitatory input; Figure 5). This effectively achieves running the simulations with a noisier clock.

There was a significant positive start–stop correlation for all target intervals (*r* = .40, .43, and .43, respectively, all *p* = .0001, Bonferroni corrected for nine comparisons). There was a significant start–spread negative correlation for all target intervals (*r* = −.61, −.61, and −.53, respectively, all *p* = .0001, Bonferroni corrected). There was a significant negative middle–spread correlation only for the second target interval (*r* = −.13, *p* = .05, Bonferroni corrected). These values were −.11 and −.01 for the short and long intervals, respectively. The CVs of start times (.18, .19, and .18, respectively) were consistently higher than those for stop times (.11, .11, and .11). Overall, these results are consistent with those of the first simulation with a lower diffusion coefficient.

### Generalizability of the results to other parameter values and observations

Different parameter values were tested to test the generalizability of the core results gathered above. Table 2 presents the overall outcomes of these simulations. The corresponding graphs (matched by model numbers) are provided in the supplemental online material. One of the key conclusions regarding the number of uncoupled TopDDM processes is that it does not materially affect the results over a wide range, provided the response threshold values are inversely related to the number of processes (see the outcomes of Model 5 and Model 6). When the response threshold is set to its original value with a low number of clock processes (e.g., *N* = 10), the activation level does not reach the threshold required to activate timed responding. With a high number of clock processes (e.g., *N* = 40), the activation level remains above the response threshold for extended periods, leading to sustained responding. Table 3 presents the baseline parameter values used in model implementations.

**TABLE 2:** The outcome of the simulations conducted with different parameter values.

Baseline parameter values						
Parameter name	Tested values	Start CV	Stop CV	Start–Stop	Start–Middle	Middle–Spread
Response threshold	10 Model 2	.15	.09	.48*	−.61*	−.17*
		.15	.09	.43*	−.66*	−.21*
		.16	.10	.47*	−.61*	−.15*
# Clocks	15 Model 3	.13	.12	.70*	−.43*	−.06
		.14	.11	.70*	−.50*	−.14
		.16	.11	.73*	−.57*	−.26*
	27 Model 4	.09	.04	.54*	−.47*	.01
		.10	.05	.57*	−.43*	.05
		.10	.04	.60*	−.39*	.08
	10 Model 5*	.18	.10	.55*	−.56*	−.13*
		.17	.09	.56*	−.58*	−.16*
		.18	.09	.62*	−.56*	−.18*
	40 Model 6*	.10	.06	.37*	−.63*	−.12
		.10	.06	.36*	−.61*	−.08
		.10	.06	.46*	−.50*	.04
Leak weight	.05 Model 7	.11	.04	.64*	−.56*	−.19*
		.11	.04	.66*	−.55*	−.20*
		.11	.04	.67*	−.54*	−.18*
	.2 Model 8	.14	.12	.77*	−.41*	−.08
		.13	.11	.71*	−.42*	−.05
		.14	.12	.69*	−.44*	−.06
Diffusion scaling	1 [BET] Model 9	.10	.05	.62*	−.59*	−.22*
		.10	.05	.62*	−.59*	−.22*
		.10	.05	.62*	−.58*	−.20*

*Note*: Parameter values that were kept constant unless indicated otherwise: Drift Rate: .20, .40, .80; Clock threshold: 10; Spike weight: 1; Response threshold: 9 (except for Models 2, 5, 6). A Bonferroni correction was applied across nine comparisons for each model. For Models 4 and 5, the response thresholds were set to 4.5 and 18, respectively, to demonstrate the relation between the number of clocks and the response threshold.

**TABLE 3:** Baseline parameter values.

Parameter name	Tested values	Start CV	Stop CV	Start–Stop	Start–Middle	Middle–Spread
Diffusion Scaling	1 [BET] Model 11	.11	.09	.31*	−.45*	.25*
		.10	.07	.24*	−.54*	.16*
		.10	.08	.42*	−.34*	.31*
	1.527 Model 12	.16	.14	.22*	−.54*	.17*
		.16	.12	.33*	−.47*	.18*
		.16	.10	.35*	−.51*	.11
Leak weight	.05 Model 13	.11	.09	.27*	−.26*	.57*
		.11	.07	.28*	−.29*	.52*
		.11	.08	.22*	−.25*	.64*
	.20 Model 14	.13	.12	.84*	−.39*	−.13
		.15	.13	.70*	−.40*	−.02
		.16	.14	.70*	−.42*	−.04
# Clocks	15 Model 15	.16	.15	.75*	−.38*	−.03
		.15	.14	.63*	−.45*	−.02
		.16	.14	.42*	−.50*	.06
	27 Model 16	.10	.07	.29*	−.39*	.36*
		.10	.06	.31*	−.39*	.33*
		.10	.06	.35*	−.31*	.42*

*Note*: Parameter values that were kept constant: Drift rate: .20, .40, .80; Clock threshold: 10; Spike weight: 1; Response threshold: 10; Logistic function steepness = 1.5.

### The probabilistic extension of the model

In the simplest version of the model, responses are emitted while the motor layer's activity level (a leaky integrator) remains above a response threshold (akin to a deterministic on–off switch). Rather than employing a hard on–off switch‐like threshold for response emission, the probabilistic extension model maps the continuous activation level of the motor leaky integrator to a momentary step‐by‐step probability of responding via a continuous logistic sigmoid function. In the extended model, the threshold parameter (motor threshold) no longer acts as a rigid execution gate. Instead, it serves as the inflection point of a smooth probability curve. This probabilistic formulation establishes a direct connection to packet theory (Kirkpatrick, 2002) and Sanabria's response‐bout frameworks (e.g., Brackney & Sanabria, 2015) in that response generation is directly tied to the instantaneous activity level of the motor‐integration layer. When the leaky‐integrator activity, *Y*(*t*), is low, the probability of responding is very low. As input spikes from Layer 1 accumulate and drive *Y*(*t*) past θ, the response probability climbs smoothly toward its upper asymptote. The sustained state of high activation of the motor integrator by the threshold crossings of uncoupled TopDDM channels over consecutive time steps, produces bouts of high‐rate responding separated by periods of pauses.

In the extended version of the ML‐DDM, the response emission is a logistic function of the instantaneous activation level of the leaky motor integrator (compared against the motor threshold). This dynamic response probability is defined as a logistic function (Equation 5). In the extended version of the ML‐DDM, there is an additional free parameter that controls the steepness of the logistic function mapping the motor layer's activity level to responses. Figure 6 shows the outcome of the simulation of the extended ML‐DDM.

(5) $$p_{\text{respond}}\left(t\right)=\frac{1}{1+e^{-\kappa \cdot \left(Y_{t}-\theta \right)}},$$

where, κ is the steepness parameter and θ is the response threshold, which corresponds to $p_{\text{respond}}=.50$.

**FIGURE 6:**
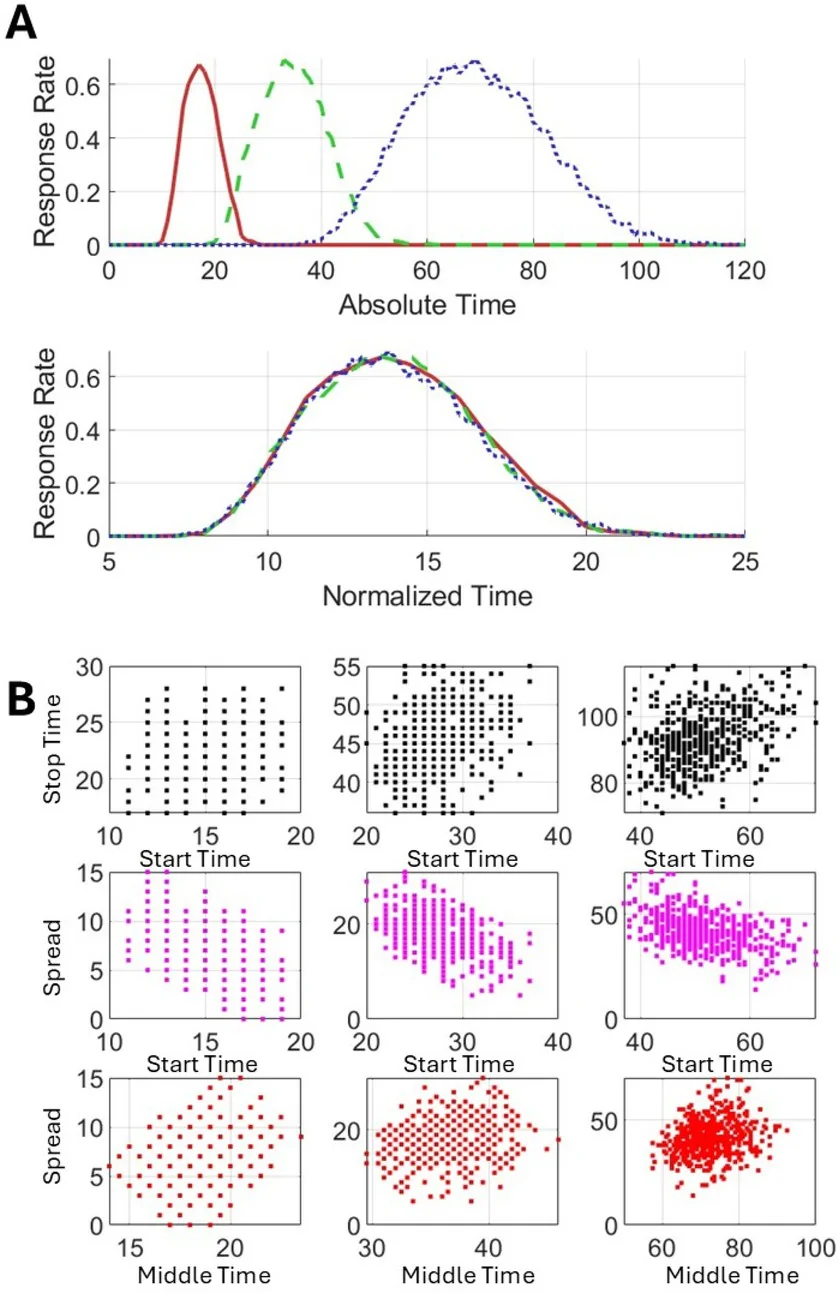
Results of simulations conducted for the extended ML‐DDM. The baseline values for Model 1 (the reduced model) were used in these simulations.

There was a significant positive start–stop correlation for all target intervals (*r* = .23, .32, and .36, respectively, all *p* = .0001, Bonferroni corrected for nine comparisons). There was a significant start–spread negative correlation for all target intervals (*r =* −.52, −.50, and −.44, respectively, all *p* = .0001, Bonferroni corrected). There was a significant middle‐time–spread positive correlation (*r =* .21, .15, and .21, respectively, *p* < .01, Bonferroni corrected). The CVs of start times (.12, .13, and .13, respectively) were consistently higher than those for stop times (.098, .087, and .091, respectively). These results more strongly support the empirical correlational patterns reported in both Luzardo et al., 2017 and Gibbon and Church, 1990, including the positive or nonsignificant correlation between the middle time and spread as well as the scalar property (Figure 6).

### Adaptation of the model to the switch time task

The model is extended to test its applicability to the timed switching task (Balci et al., 2008, 2009; Fetterman & Killeen, 1995). Unlike the ML‐DDM formulated above, the application of the model to the switch task involved two sets of TopDDM processes that were active simultaneously, each set timing the intervals associated with its options. The threshold first‐crossing time of the processes broadcast from their own dedicated pool of independent TopDDM processes is accumulated by the corresponding motor layer unit with scaled leakage (the baseline leak rate of .10 was used, and the response threshold was set to 5). Crucially, mutual inhibition between the two motor units is introduced (Equation 6) to negotiate responses between two timed options according to the leaky competing accumulator model of Usher and McClelland (2001). This implementation of the competitive selection mechanism largely prevents the cotriggering of conflicting behaviors.

(6) $$\frac{dY_{j}\left(t\right)}{\mathrm{dt}}=\omega \sum_{i=1}^{N_{j}}\delta \left(t-t_{i,j}\right)-\alpha A_{j}\cdot Y_{j}\left(t\right)-\beta \sum_{k\neq j}Y_{K}\left(t\right),$$

subject to $x_{i,j}\left(t_{i,j}\right)\geq z$, where $t_{i,j}$ is the threshold‐crossing time of the *i* ^th^ TopDDM process broadcasting to channel *j* and β is the positive scaling factor representing the strength of the mutual inhibition between competing motor units. $Y_{K}\left(t\right)$ is the instantaneous internal state of the rival motor integrator. The explicit mutual inhibition portion $-\beta \sum_{k\neq j}Y_{K}\left(t\right)$ subtracts from the net input of the integrator. As a result, when there is a highly reliable time‐dependent broadcast from Layer 1, the corresponding motor unit activity increases quickly, thereby actively suppressing the accumulation of activity in the competing channel. This favors the short option unit early in the trial and the long option unit later (Figure 7). The extension of the model to the timed switch task includes an additional free parameter that determines the degree of inhibition each motor unit exerts on the other based on their activity levels.

**FIGURE 7:**
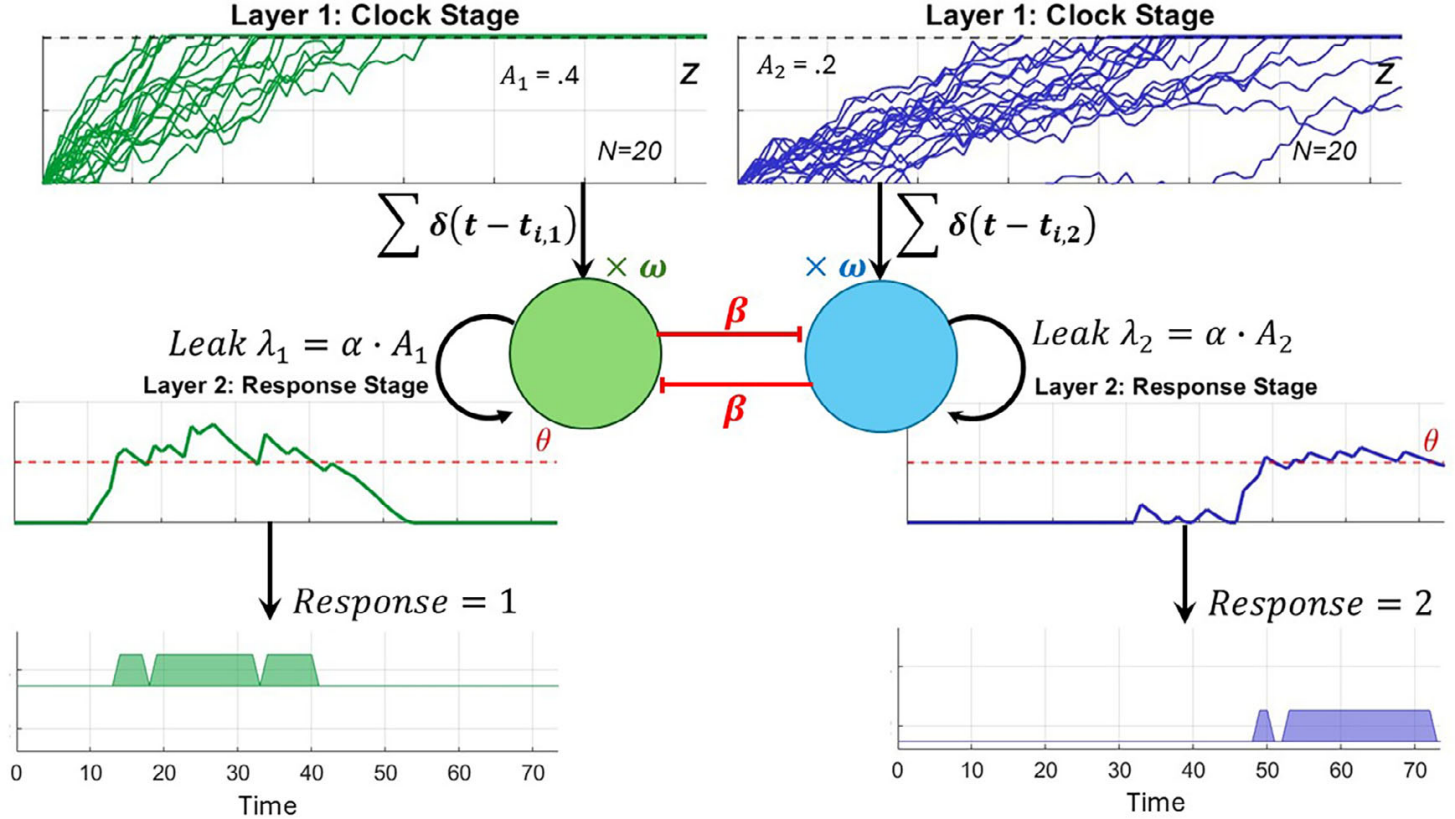
Illustration of the version of ML‐DDM applied to the switch task (Balci et al., 2008, 2009) with mutual inhibition between the motor units.

The switch time distributions represented the empirical data (Figure 8A–B; Balci et al., 2008, 2009; Fetterman & Killeen, 1995). In support of the scalar variability, the CVs of switch times from simulations using drift rates of .20 versus .40 and .40 versus .80 were virtually identical, at .044 and .041, respectively.

**FIGURE 8:**
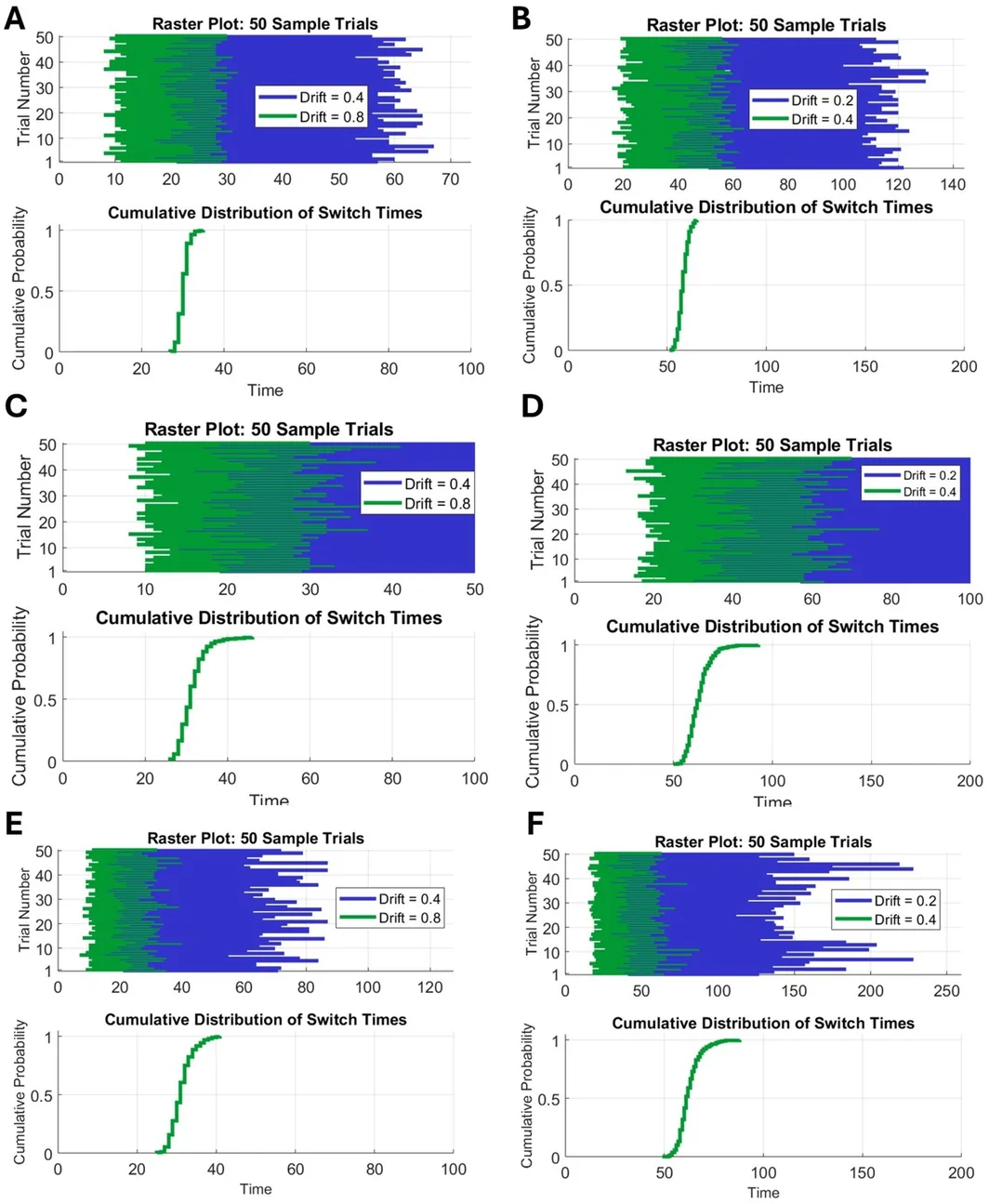
The emergent raster plots and the cumulative distribution of the switch times for the deterministic on–off switch‐like response rule (A–B), probabilistic (logistic) response rule (C–D), and probabilistic response rule with a lower required activation at the response threshold (E–F).

The same model was simulated using a logistic function that mapped the activation level of the motor layer to the response probability, which closely represented the empirical data (Figure 8C–D). In support of scalar variability, the CV values were .098 and .091 for the shorter and longer pairs of timing options, respectively. When a lower required activation level is imposed (i.e., effective response probability starts at .50 at the motor threshold), the CV values were .087 and .089 for the shorter and longer pairs of timing options, respectively (Figure 8E–F).

## DISCUSSION

This article presents a new model of timing behavior in the PI procedure grounded in the TOPDDM. Different from our earlier TopDDM‐based models, the functional architecture of this model comprises two layers (but see Akdoğan & Balcı, 2017; Balcı & Simen, 2016; Balcı & Öztel, 2026). While the first layer implements TopDDM, adhering to all its core assumptions, including the assumption of a single, maximally set threshold to optimize temporal precision, it differs from TopDDM's original implementation. In the original implementation of TopDDM (Balcı & Simen, 2016; Simen et al., 2011, 2013), a single integration process is assumed to underlie the point‐timing behavior. In ML‐TopDDM, multiple uncoupled drift‐diffusion processes are assumed to be manifested in parallel. Critically, TopDDM assumes that the timing behavior is automatically triggered by the TopDDM's threshold crossings; if responses are emitted at each threshold crossing, a unimodal distribution (i.e., an inverse‐Gaussian distribution) would result when multiple TopDDMs are in effect in parallel. This prediction clearly contradicts the break‐run‐break pattern observed in the animals' responses across individual trials. Note that the ML‐DDM model preserves the original logic of TopDDM by not compromising Layer 1; it keeps its threshold fixed at the highest level to maximize precision.

The ML‐TopDDM breaks this property of TopDDM by assuming that each temporal accumulation's threshold crossing broadcasts a spike to a downstream motor layer, which integrates these signals with a leak. When the accumulated amount of signals reaches a high‐set response threshold (θ), the animal is assumed to initiate and continue responding while the value remains above this threshold. How this motor‐layer activity is mapped onto timing responses depends on the nature of the activation function linking the internal state to behavior. In this article, both the deterministic on–off switch rule and sigmoidal rule were used. For practical purposes, both rules yield desirable results.

Critically, the leak rate is assumed to be scaled with the drift rate. Briefly, the ML‐TopDDM's second layer acts as a time‐decaying weighted memory of all past evidence that “the reward time has reached.” For a sufficiently high response threshold, this property of the model prevents emission of responses based on an outlier threshold crossing (due to the noise component). This important property of the scaled leak rate is adaptive, ensuring that the signal‐to‐noise ratio remains constant across different target times, which results in timescale invariance of the motor layer's outputs. The use of different leak values for different timescales is not new; this property was also used by Simen et al. (2011), who assumed chains of leaky integrators that had a decreasing sequence of intrinsic time constants to implement feedback‐based integrators—namely, lower drift rates naturally forced to rely on longer leak‐time constants (smaller leak values) to maintain the integration state over extended periods.

Within the two‐alternative forced‐choice decision framework, the relation between the drift rate and the leak rate helps optimize the signal‐to‐noise ratio. Since the threshold‐crossing times are highly uncertain and subject to substantial temporal jitter, the downstream integrator must extract a clean signal from these noisy dynamics, which it achieves by averaging broadcast information over a wide temporal window. Within this framework, the downstream leaky integrator serves as a temporal smoothing filter (Verdonck et al., 2020). This also aligns well with a normativity account, as when the environment is variable or the evidence strength is unstable within a trial, the ideal observer should adjust the integration window dynamically so that it can afford high leak when the evidence strength is strong, prioritizing speed. On the other hand, when the evidence strength is very low, the observer should make use of every bit of evidence over time to drive the motor system toward the correct boundary.

The current model is similar to the leaky integrating threshold (LIT) model (Balsdon et al., 2023; Verdonck et al., 2020) in that it separates the sensory decision variable from the motor execution system via a two‐layer cascade in which evidence for both choices is processed at each layer. However, unlike the current model, in LIT the evidence at the primary sensory layer is represented using a standard drift‐diffusion model, with a single continuous decision variable that reflects the relative difference in evidence. However, unlike the conventional DDM architecture and ML‐TopDDM, in LIT the decision variable continuously drifts and diffuses during the trial; the system is assumed to be threshold free at the primary sensory layer. Again, different from these models, LIT converts the directional value of the sensory evidence into two distinct motor actions by splitting the motor layer into two independent competing leaky integrators that only listen to when the evidence favors the choice represented by a given motor layer, allowing the “simultaneously” ramping up behavior by both motor plans (akin to electromyography activity being observed in both hands before the choice). The choice is made based on which independent threshold of the motor stage is hit first.

This class of models brings a novel approach to linking perceptual/temporal evidence to choice and timing behavior in the PI procedure. The standard forms of the drift‐diffusion model and its time‐adaptive opponent‐Poisson variant assume that behaviors are emitted upon crossing the threshold. The ML‐DDM, as well as LIT, provides a more dynamic view in that it relies on secondary motor integration playing a clear role in sensorimotor decision making and timing behavior. This new approach attributes a more integral role to the premotor system in decision making (e.g., Thura & Cisek, 2016) and interval timing, capturing the effect of both sensory and temporal accumulation on motor planning. The neural evidence supporting the clock‐adaptive temporal accumulation has already been widely reported (Bruce et al., 2025; Merchant & Averbeck, 2017; Emmons et al., 2020; Jazayeri & Shadlen, 2015; Komura et al., 2001; Leon & Shadlen, 2003; Macar & Vidal, 2003; Murakami et al., 2017; Ofir & Landau, 2022), whereas the LIT‐like motor accumulation process has been reported by Balsdon et al. (2023). The two‐layer functional architecture presented in this article may serve as a conceptual benchmark for evaluating neural data (e.g., Ofir & Landau, 2022).

The version of the model that mapped motor‐layer activity level to response probability performed better than the original version with a deterministic, on–off, switch‐like rule. This leverage of the probabilistic response function was primarily based on its accounting of the positive middle‐time–spread correlation (according to benchmarks set in Luzardo et al., 2017). The model implementations also supported the higher CVs of start times than the stop times and their constancy across durations within a model. The probabilistic response function also aligns more closely with prior theoretical developments in interval timing (Brackney & Sanabria, 2015; Kirkpatrick, 2002). The extension of the model to the switch task with a mutual‐inhibition component resulted in an empirically similar distribution of switch times and a constant CV across different duration pairs. Notably, all model implementations strongly supported the scalar property of interval timing, whether through the superposition of average response curves or through the near‐constant CVs of start time, stop time, and switch time.

There are other benchmarks of the PI procedure that may be considered in further extensions of the model. For example, a critical benchmark of the PI procedure is the phenomenon of response resurgence (continued responding well after the reward is omitted; see Sanabria & Killeen, 2007). This can be accounted for within the framework of ML‐DDM by adjusting the dynamics of Layer 1 so that the timers, or a subpopulation of them, reset to 0 when the corresponding temporal particles hit the timing threshold. This would allow a single process to fire multiple times over a long trial. Under an extended probe, a secondary wave of rhythmic or stochastic alignment can cause the integrator to climb back up later, capturing Sanabria and Killeen's (2007) resurgence phenomenon.

Another important issue worth noting is that the model assumes the drift rate (*A*) is a function of the target interval (*T*), a consequence of the steady‐state focus of the current work and not of the clock's adaptive learning during the trial. In other words, the model architecture does not contain a learning component. The early and late learning rules formulated by Simen et al. (2011) and Rivest and Bengio (2011) can be applied directly to parameterizing the drift rate in Layer 1.

A central feature of the ML‐DDM is that the motor layer's leak rate (𝜆) scales with the drift rate (*A*) to optimize the signal‐to‐noise ratio. This is a necessary property of the model to achieve scalar variability (akin to the balanced excitation/inhibition in TopDDM; Balcı & Simen, 2016; Simen et al., 2011). Although this may appear as a “baked‐in” desired outcome, as argued earlier, it is grounded in a normative framework that specifies *what* the system must do to maintain optimality (see also Glaze et al., 2015). Furthermore, this core relation may directly emerge from simple, biologically plausible network dynamics. For example, when the input rate increases, it opens more synaptic channels, thereby increasing network conductance and, as a biophysical consequence, increasing the leak rate (reducing resistance; Chance et al., 2002; Dayan & Abbott, 2001). From a purely functional perspective, a lower input rate increases the working memory load (in parallel with the integration of Layer 1's broadcast signals by Layer 2), which the system addresses by reducing the rate at which information decays (corresponding to higher working memory load).

Although ML‐DDM introduces a novel multiprocess architecture, it builds on a rich historical framework of leaky integration and multiclock architectures in modeling interval timing. For example, the conceptualization of temporal accumulation via leaky processes was formalized by Staddon (2005) in multiple‐time‐scale models, where memory traces decay over time to track intervals rather than relying on accumulators. Furthermore, the use of parallel processes aligns with the “multiple‐clock” tradition. This includes early models of memory‐driven, parallel‐decay timing mechanisms (Staddon & Higa, 1996) as well as more contemporary computational approaches that assert that the brain maintains a set of timescales or multirate neural clocks to represent the temporal statistics of the environment (Gershman, Radulescu, et al., 2014). Crucially, this multiscale representation of time has been map‐matched to neural substrates; for example, Gershman, Moustafa, et al. (2014) demonstrated how parallel temporal representations underwrite reinforcement‐learning computations within the basal ganglia, providing a biological precedent for multichannel temporal architectures. By combining multiple parallel tracking channels with a subsequent leaky‐convergence stage, ML‐DDM bridges classic multiclock frameworks and basal ganglia implementations to contemporary accumulator theories.

Future work may extend the scope of ML‐DDM to model behaviors in other tasks, such as temporal generalization (e.g., Church & Gibbon, 1982). The effects of reward magnitudes and probabilities may be implemented at the level of the value of spike weight (ω) or the degree of asymmetric inhibition (κ), where the value may be higher for a higher probability and/or higher magnitude option.

## AUTHOR CONTRIBUTIONS

Fuat Balcı is the sole author of this article.

## CONFLICT OF INTEREST STATEMENT

I declare no conflict of interest relevant to the research.

## ETHICS APPROVAL

This paper is theoretical. Thus, no ethical approval was required.

## Supporting information


**Data S1**: Supporting Information

## Data Availability

This paper is theoretical, and no new data were collected.
