# Supplementary material for: A multilayer drift‐diffusion broadcast model: Converting temporal evidence into response timing
Source: J Exp Anal Behav. 2026 Oct 26;126(3):e70150. doi: 10.1002/jeab.70150 (PMC13710114; doi:10.1002/jeab.70150)
Supplement: Supplementary file 1 [file JEAB-126-0-s001.zip › 3_Balci_Supplemental Material_e.pdf]

MODEL 2

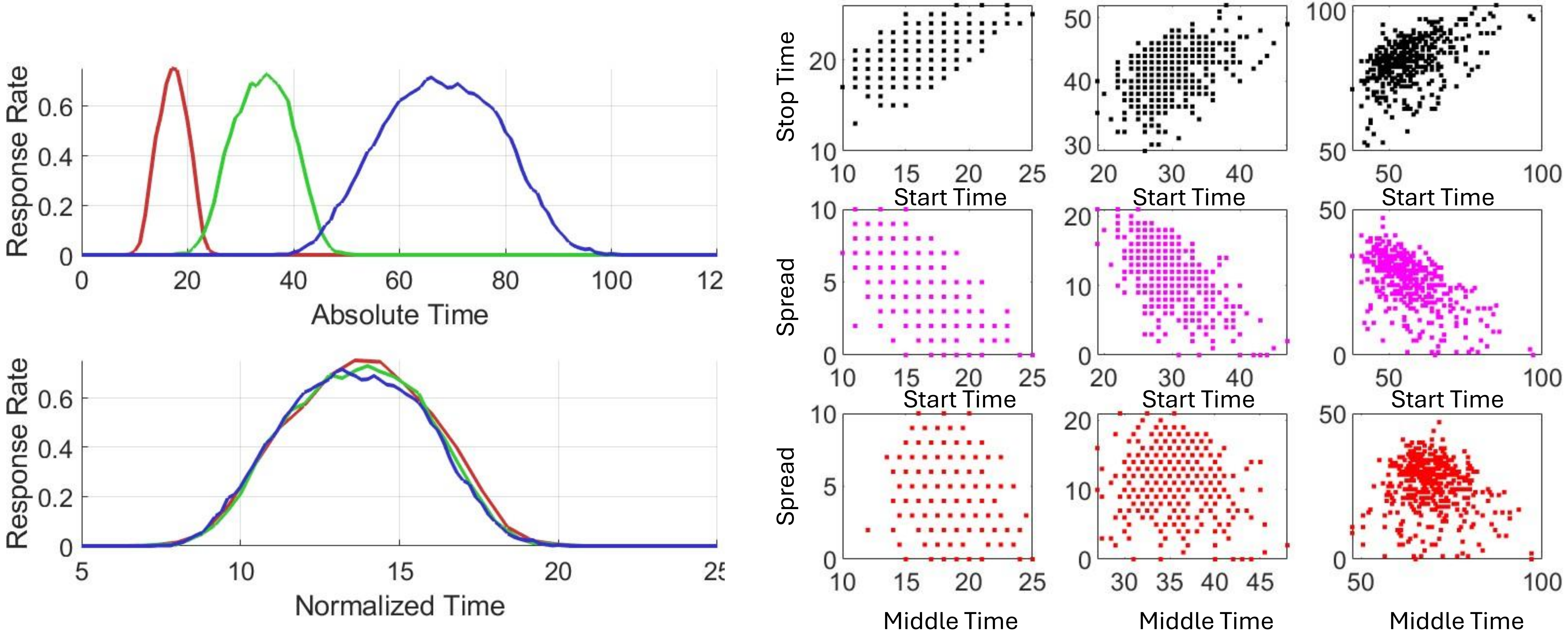

Note: Baseline with Response Threshold = 10

MODEL 3

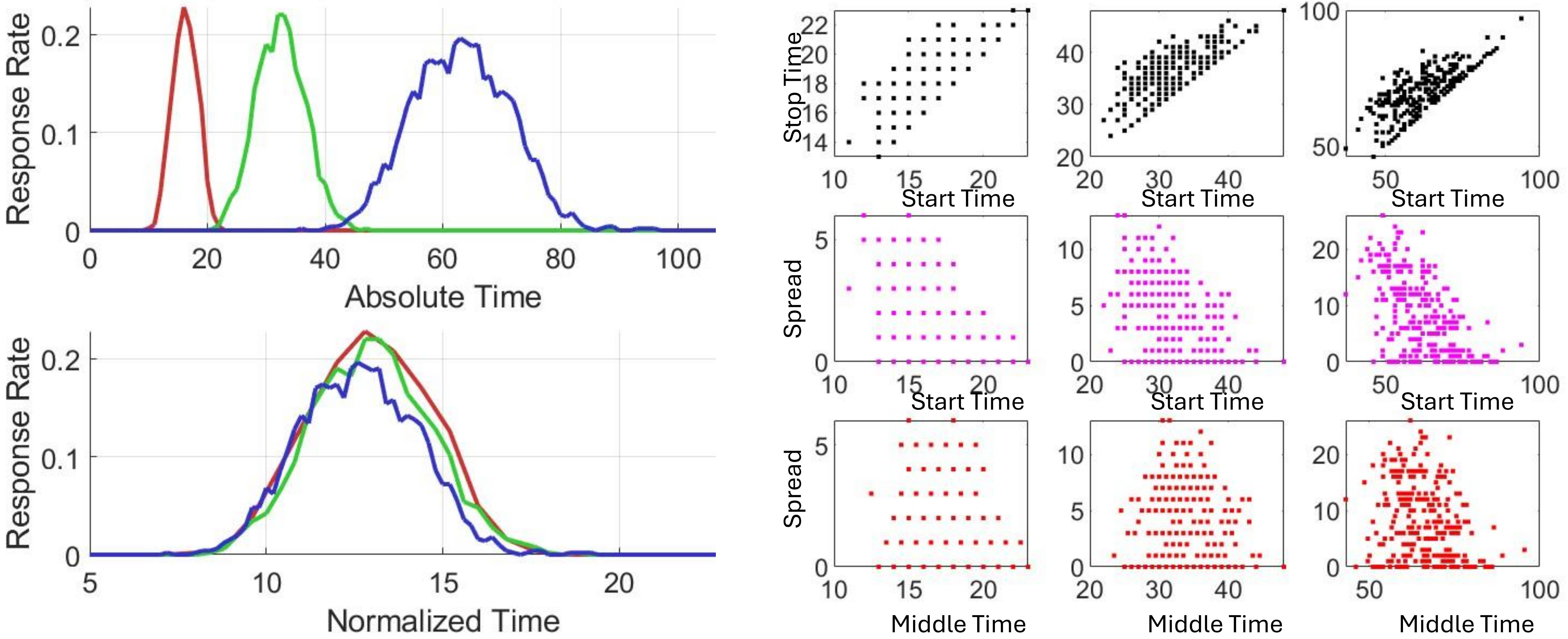

Note: Baseline with # Clock = 15

MODEL 4

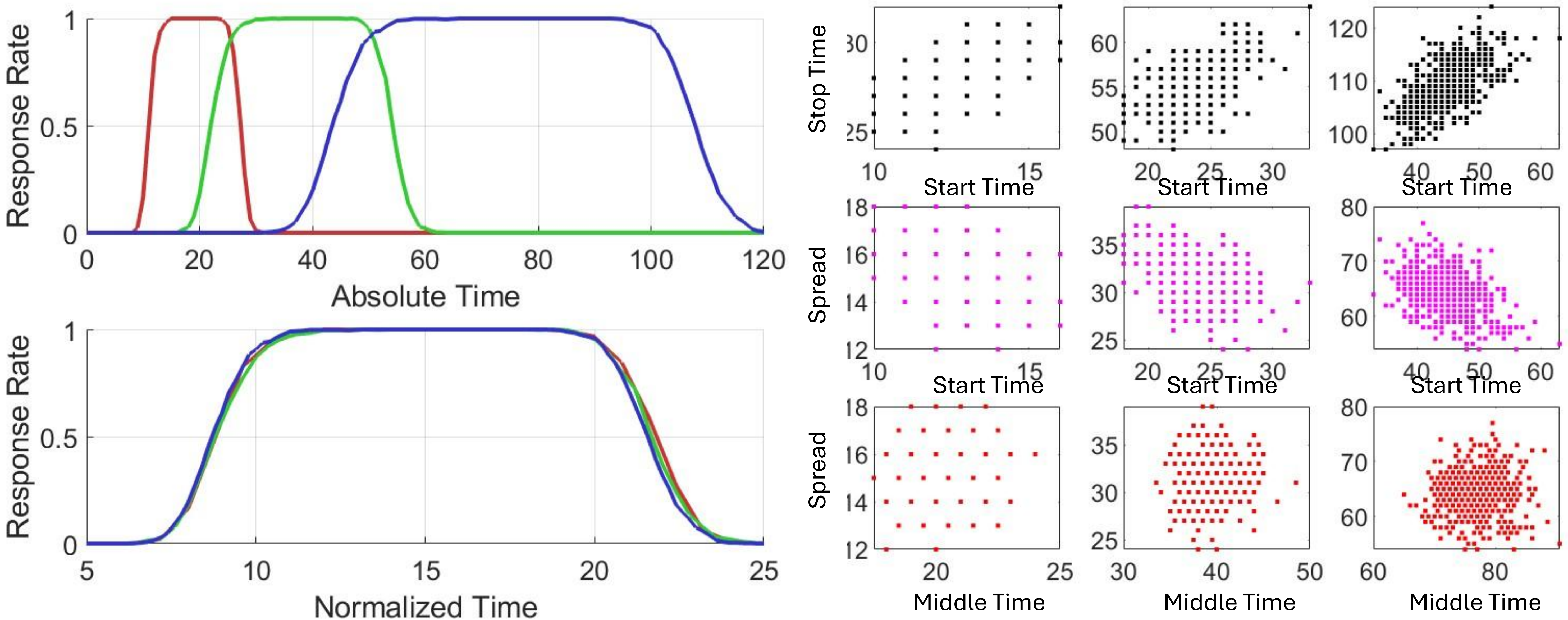

Note: Baseline with # Clock = 27

MODEL 5

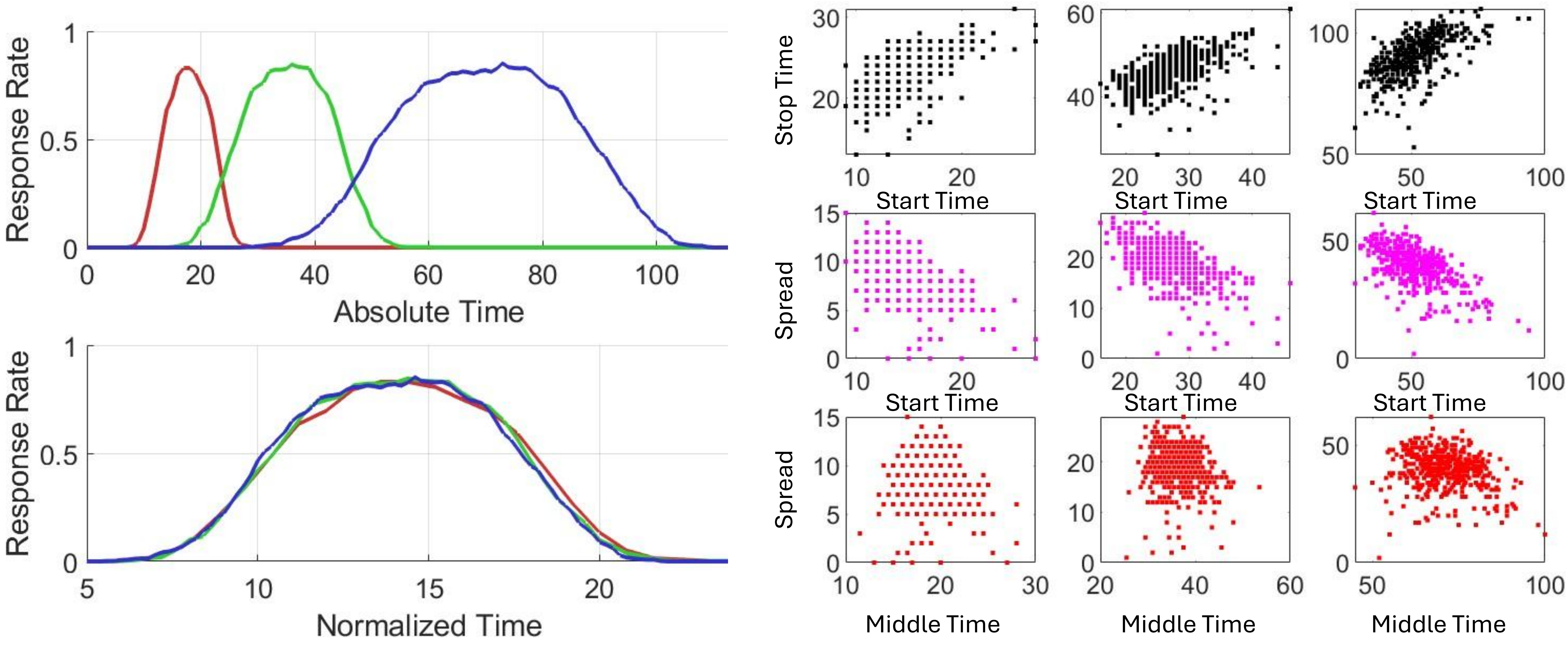

Response Rate
Absolute Time
Response Rate
Normalized Time
Stop Time
Start Time
Spread
Start Time
Spread
Middle Time

MODEL 6

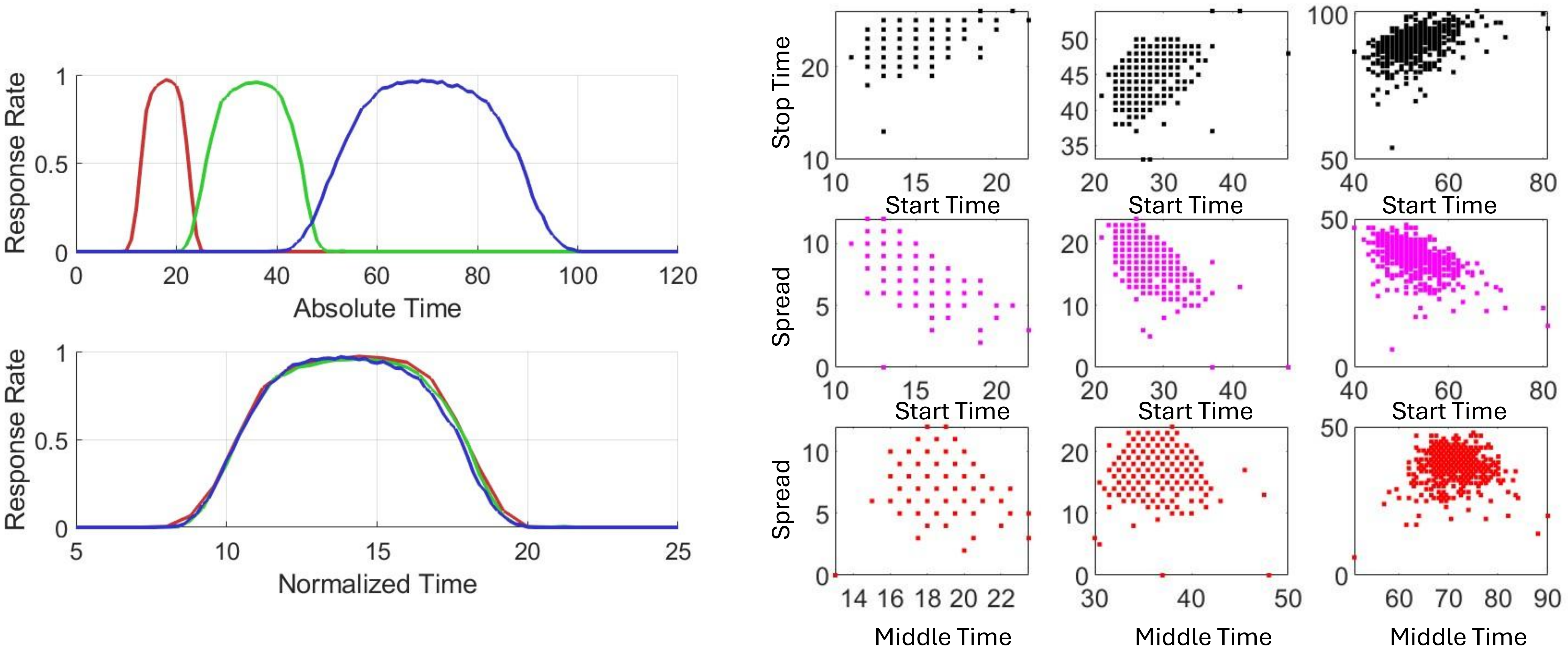
Response Rate
Absolute Time
Normalized Time
Stop Time
Start Time
Spread
Middle Time

MODEL 7

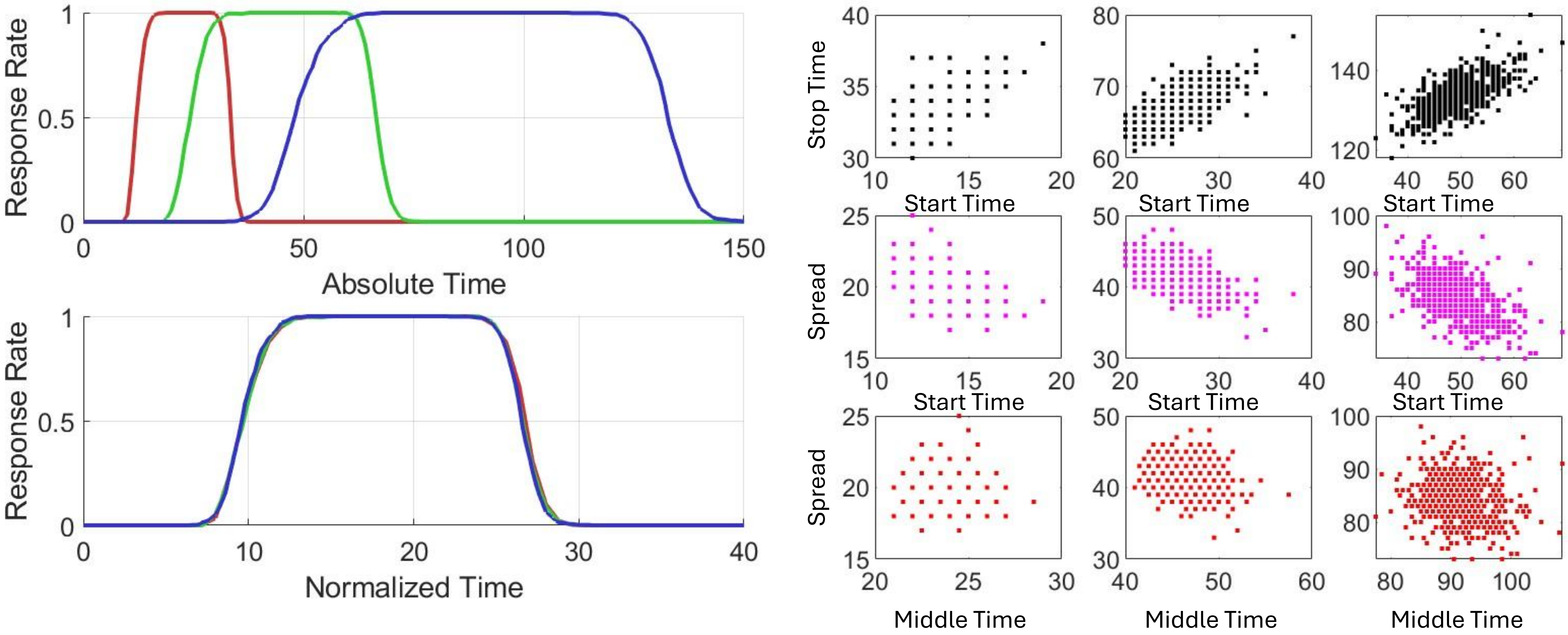

Response Rate
Absolute Time
Response Rate
Normalized Time
Stop Time
Start Time
Spread
Start Time
Spread
Middle Time

MODEL 8

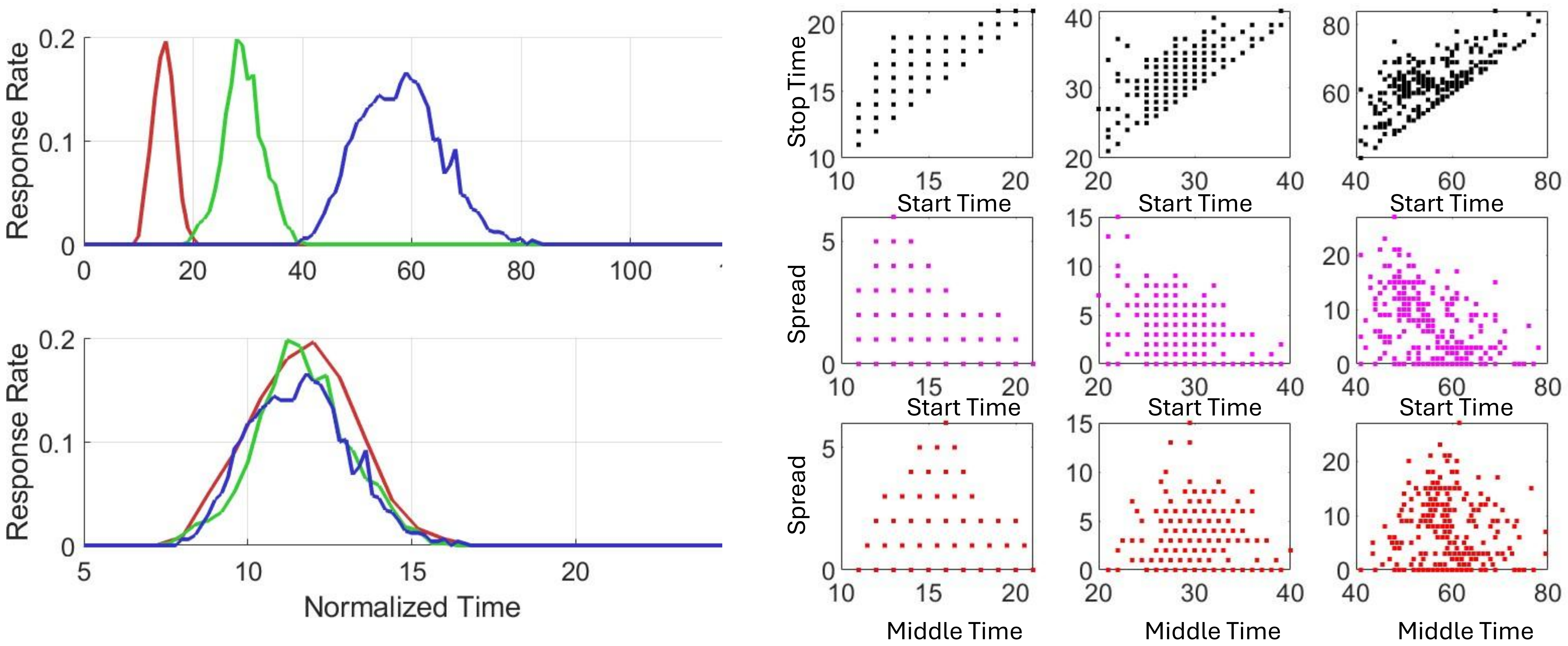

Response Rate
0.2
0.1
0
0
20
40
60
80
100
Response Rate
5
10
15
20
Normalized Time
Stop Time
Start Time
Spread
Start Time
Spread
Middle Time

MODEL 9

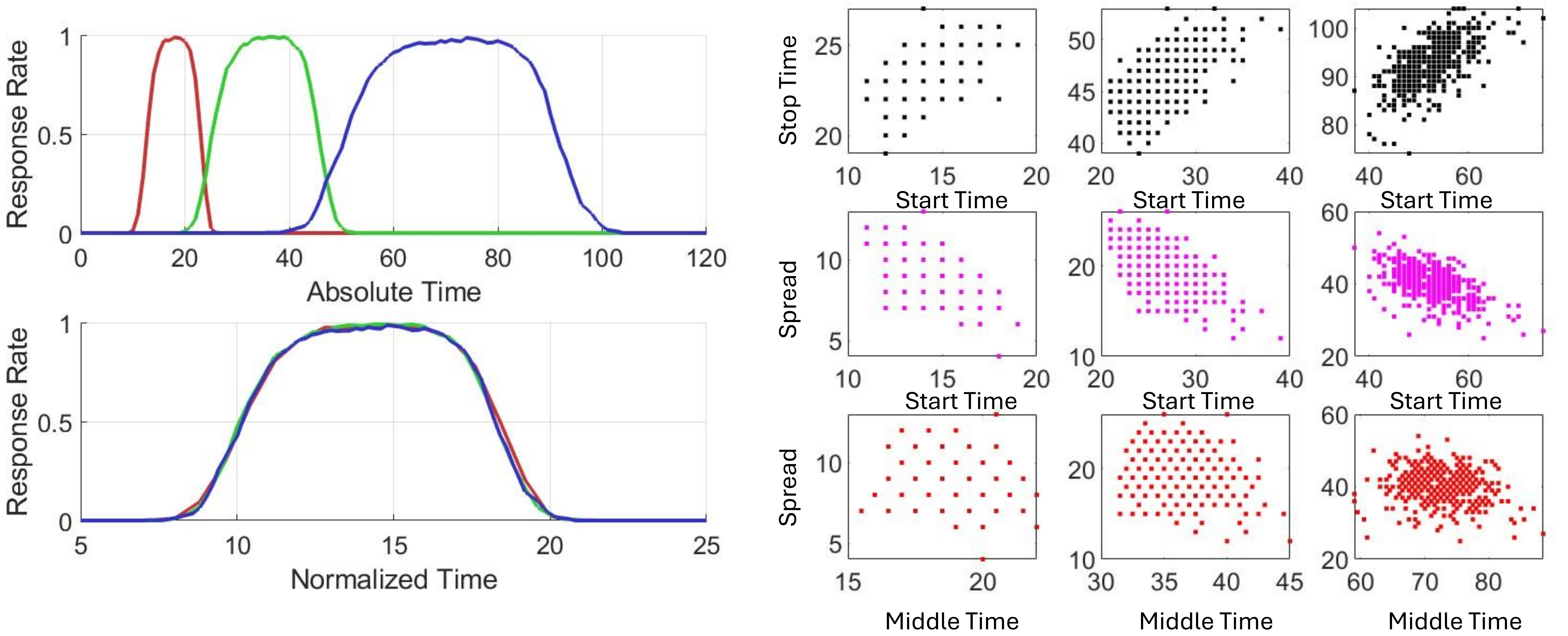
Response Rate
Absolute Time
Response Rate
Normalized Time
Stop Time
Start Time
Spread
Start Time
Spread
Middle Time

MODEL 11

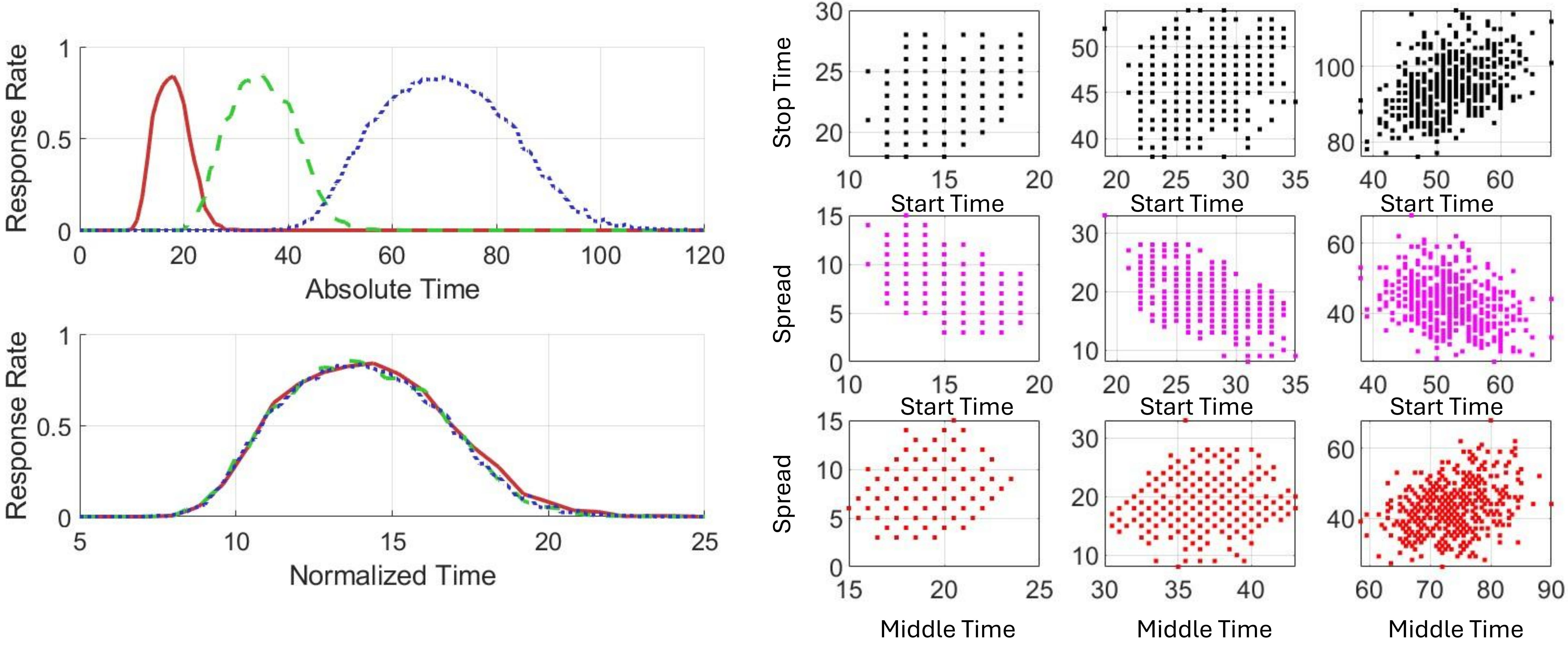

Response Rate
Absolute Time
Response Rate
Normalized Time
Stop Time
Start Time
Spread
Start Time
Spread
Middle Time

MODEL 12

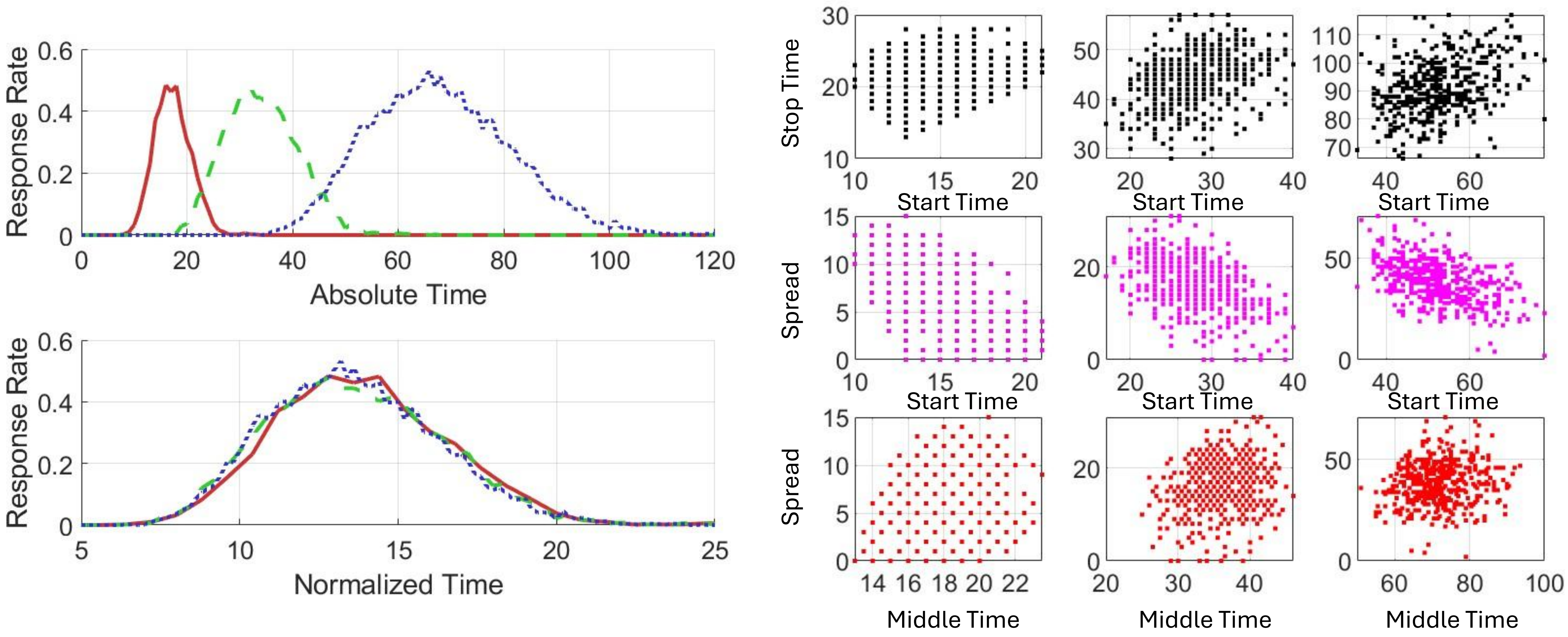

Response Rate
Absolute Time
Response Rate
Normalized Time
Stop Time
Start Time
Spread
Start Time
Spread
Middle Time

MODEL 13

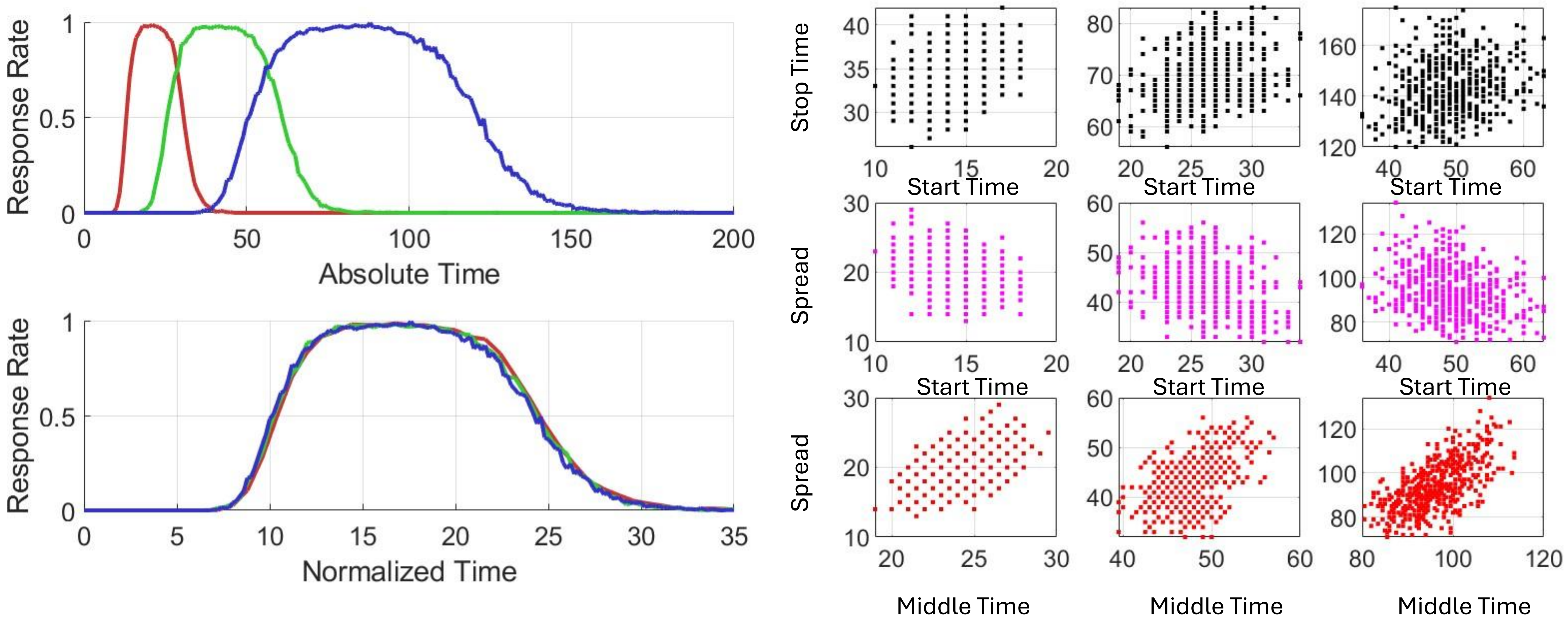

Response Rate
1
0.5
0
0
50
100
150
200
Absolute Time
Response Rate
1
0.5
0
0
5
10
15
20
25
30
35
Normalized Time
Stop Time
Start Time
Start Time
Start Time
Spread
Start Time
Start Time
Start Time
Spread
Middle Time
Middle Time
Middle Time

MODEL 14

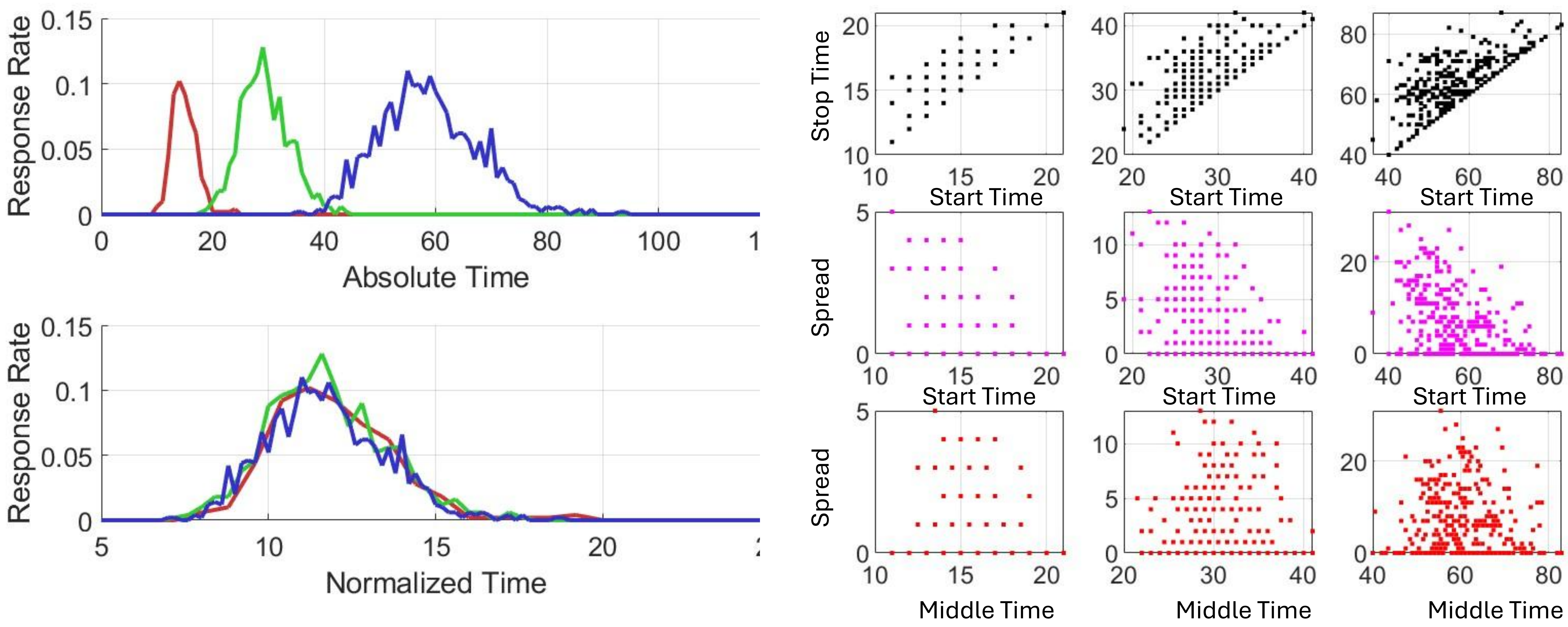

Response Rate
0.15
0.1
0.05
0
0
20
40
60
80
100
Absolute Time
Response Rate
0.15
0.1
0.05
0
5
10
15
20
Normalized Time
Stop Time
Start Time
Spread
Start Time
Spread
Middle Time

MODEL 15

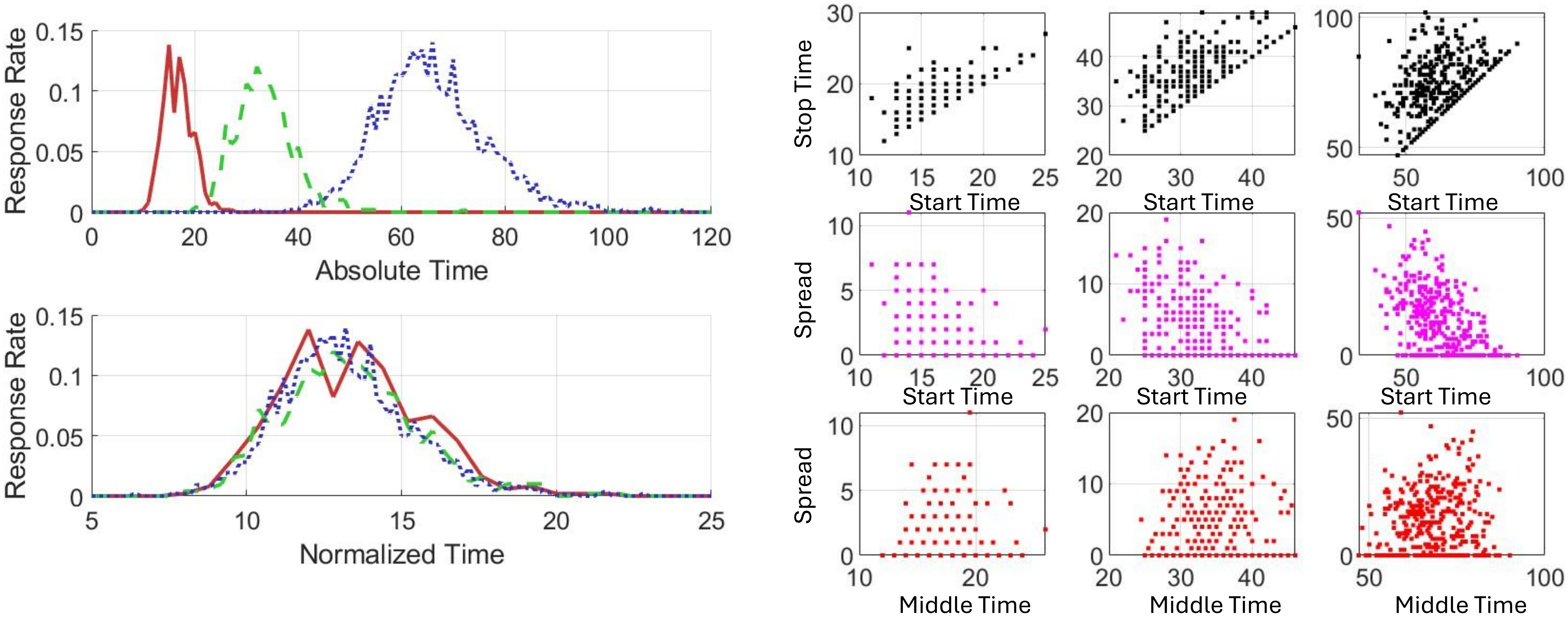

Response Rate
0.15
0.1
0.05
0
0
20
40
60
80
100
120
Absolute Time
Response Rate
0.15
0.1
0.05
0
5
10
15
20
25
Normalized Time
Stop Time
Start Time
Spread
Start Time
Spread
Middle Time

MODEL 16

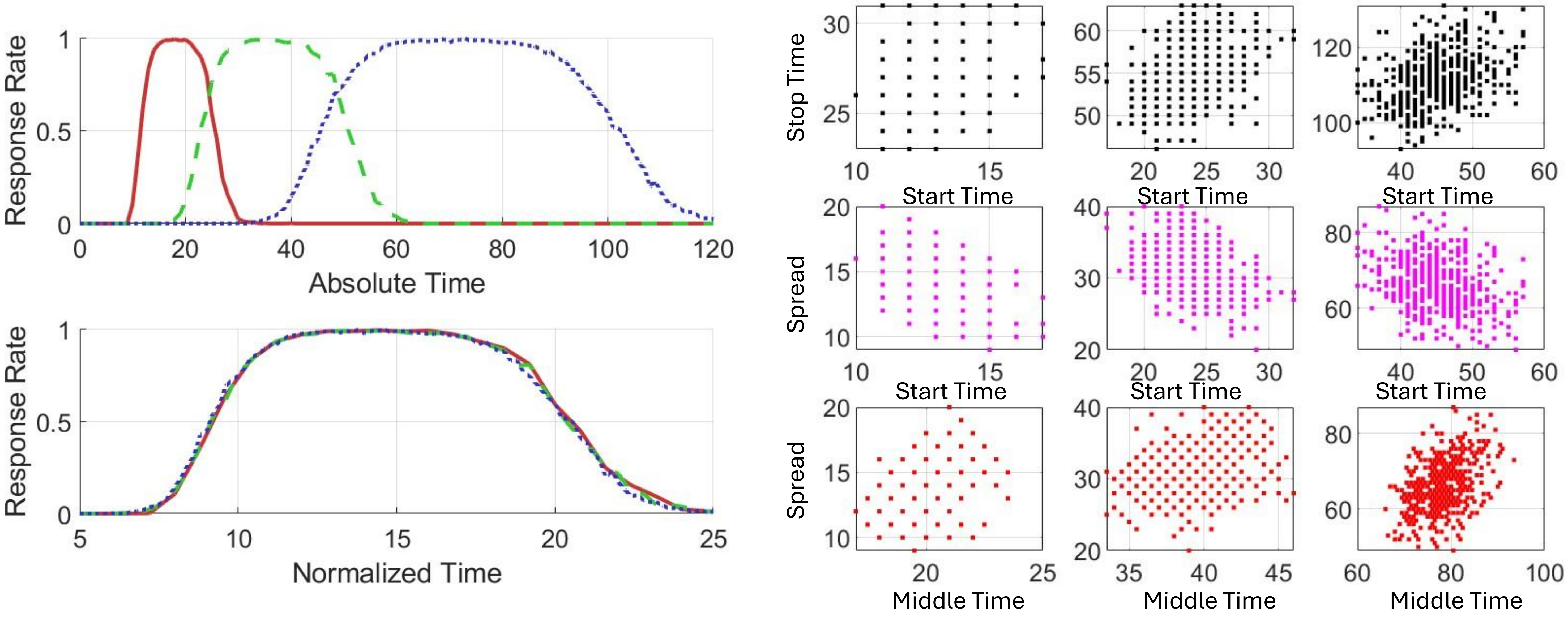

Response Rate
Absolute Time
Response Rate
Normalized Time
Stop Time
Start Time
Spread
Start Time
Spread
Middle Time
